# Home-Based Transcranial Direct Current Stimulation vs Placebo for Fibromyalgia: A Randomized Clinical Trial

**DOI:** 10.1001/jamanetworkopen.2025.14262

**Published:** 2025-06-06

**Authors:** Wolnei Caumo, Barbara Regina Franca, Roman Orzechowski, Graziele Bueno, Arthur França de Souza, Jéssica Vebber dos Santos da Silva, Paulo R. S. Sanches, Danton P. Da Silva, Iraci L. S. Torres, Vania Naomi Hirakata, Kevin Pacheco-Barrios, Felipe Fregni

**Affiliations:** 1 Post-Graduate Program in Medical Sciences, School of Medicine, Universidade Federal do Rio Grande do Sul (UFRGS), Porto Alegre, Brazil; 2 Laboratory of Pain and Neuromodulation at Hospital de Clínicas de Porto Alegre (HCPA), Porto Alegre, Brazil; 3 Pain and Palliative Care Service at HCPA, Porto Alegre, Brazil; 4 Laboratory of Biomedical Engineer at HCPA, Porto Alegre, Brazil; 5 Laboratório de Farmacologia da Dor e Neuromodulação, Investigações Pré Clínicas, Centro de Pesquisa Experimental, HCPA, Porto Alegre, Rio Grande do Sul, Brazil; 6 Unidade de Bioestatística, Diretoria de Pesquisa, HCPA, Porto Alegre, Brazil; 7 Department of Surgery, School of Medicine, UFRGS, Porto Alegre, Brazil; 8 Neuromodulation Center and Center for Clinical Research Learning, Spaulding Rehabilitation Hospital and Massachusetts General Hospital, Harvard Medical School, Boston, Massachusetts; 9 Unidad de Investigación para la Generación y Síntesis de Evidencias en Salud, Vicerrectorado de Investigación, Universidad San Ignacio de Loyola, Lima, Peru

## Abstract

**Importance:**

Previous trials with smaller samples and shorter follow-up periods showed that multiple-session home-based anodal transcranial direct current stimulation (A-tDCS) on the left dorsolateral prefrontal cortex (DLPFC) improved fibromyalgia symptoms. However, the duration of the effect, the influence of exercise and pain neuroscience education (PNE), and the role of placebo remain unclear.

**Objective:**

To evaluate whether A-tDCS targeting the left DLPFC, combined with exercise and PNE, is more effective than sham tDCS in reducing pain and disability, based on placebo-test responses (responders vs nonresponders).

**Design, Setting, and Participants:**

This double-blind, sham-controlled randomized clinical trial enrolled women aged 18 to 65 years with fibromyalgia. Participants were randomized to receive A-tDCS or sham tDCS between April 2022 and April 2024. They were treated at home and at the outpatient Clinical Research Center of Hospital de Clínicas de Porto Alegre in Porto Alegre, Brazil. Exclusion criteria included tDCS contraindications and uncontrolled clinical conditions. Intention-to-treat analyses were conducted from July to December 2024.

**Interventions:**

Home-based tDCS (2 mA; 20 minutes daily) or sham tDCS (2 mA; 30 seconds at the start, then 10 minutes, and then 20 minutes, with a 20-second ramp-up and ramp-down) for 4 weeks with anodal-left and cathodal-right prefrontal stimulation (35 cm^2^ electrodes), combined with exercise and PNE via videos and remote supervision following in-person training.

**Main Outcomes and Measures:**

Change in Multidimensional Pain Interference Index (MPII) at treatment end and 3-month follow-up. MPII was measured by the Brief Pain Inventory, a 7-item scale that assesses the impact of pain on daily activities, emotional well-being, and social interactions.

**Results:**

A total of 112 female patients (mean [SD] age, 49.04 [9.71] years) were included and randomized to either A-tDCS (n = 56 placebo nonresponders) or sham tDCS (n = 56 placebo responders). In the intention-to-treat analysis, linear mixed-effects models showed reduced MPII by 38.76% (95% CI, −41.90% to −30.92%) for the A-tDCS group vs 16.08% (95% CI, −21.42% to −10.41%) for sham tDCS (mean difference [MD], 22.68%; 95% CI, 12.79%-40.00%; Cohen *d* = 0.73). A significant treatment-by-time interaction favored A-tDCS across 5 assessments, with no interaction by placebo response. In placebo responders, MPII was reduced by 34.21% (95% CI, −46.88% to −28.29%) for A-tDCS vs 18.13% (95% CI, −24.90% to 3.34%) for sham tDCS (MD, 24.23%; 95% CI, 15.80%-32.67%). Among placebo nonresponders, MPII decreases were 35.49% (95% CI, −41.21% to −29.53%) for A-tDCS vs 25.96% (95% CI, −34.31% to −20.42%) for sham tDCS (MD, 9.52%; 95% CI, 2.79%-19.78%). Improvement in MPII of 50% or more was achieved by 62.5% of participants (n = 35) in the A-tDCS group vs 37.5% (n = 21) in the sham tDCS group (relative risk, 0.60; 95% CI, 0.39-0.91).

**Conclusions and Relevance:**

This trial found that A-tDCS along with exercise and PNE improved disability due to pain, especially in placebo test responders. The findings support fibromyalgia management and enhance understanding of tDCS-related placebo effects.

**Trial Registration:**

ClinicalTrials.gov Identifier: NCT05845528

## Key Points

**Question:**

Is a home-based, self-applied anodal transcranial direct current stimulation (A-tDCS) to the left dorsolateral prefrontal cortex, combined with exercise and pain neuroscience education (PNE), effective for treating pain and pain-related disability, considering placebo-response propensity?

**Findings:**

In this randomized clinical trial involving 112 women with fibromyalgia, A-tDCS combined with exercise and PNE significantly outperformed sham tDCS with exercise and PNE. The treatment demonstrated clinically and statistically significant reductions in pain severity, improved pain-related disability, and enhanced endogenous pain modulation, particularly among placebo responders.

**Meaning:**

The findings indicate that A-tDCS with exercise and PNE may be an effective clinical approach to managing fibromyalgia symptoms and understanding tDCS-related placebo effects.

## Teaser

This randomized clinical trial assesses pain reduction from a self-administered intervention (transcranial direct current stimulation device) coupled with video-based exercise and pain neuroscience education for women with fibromyalgia.

## Introduction

Fibromyalgia affects 2.0% to 5.8% of the general population and is characterized by chronic pain, fatigue, sleep disturbances, and cognitive emotional symptoms.^1^ Medication adherence is low, with 72.5% of patients not following prescriptions^2^ and 67.0% of duloxetine users showing poor adherence.^3^ Moreover, drugs such as duloxetine, amitriptyline, gabapentin, and pregabalin achieve at least 50% pain relief in only 10% to 15% of patients with fibromyalgia.^4^ As a result, current guidelines emphasize nonpharmacological interventions,^5,6^ including transcranial direct current stimulation (tDCS), to improve pain and related symptoms.^7^

The tDCS modulates neuronal excitability and neuroplasticity through low electrical currents (0.5-2 mA).^8,9^ Despite its therapeutic potential, tDCS requires multiple sessions, posing challenges such as travel burdens and limited clinic hours.^10^ To overcome these barriers, home-based anodal tDCS (A-tDCS) has been validated for remote use and has demonstrated efficacy in managing fibromyalgia symptoms in studies involving 20 to 60 sessions.^11,12^ tDCS targeting the primary motor cortex has proven effective in reducing pain severity.^11,13,14,15^ However, stimulating the left dorsolateral prefrontal cortex (DLPFC) offers broader benefits, such as alleviating depressive symptoms, enhancing cognitive function, reducing pain catastrophizing, and improving disability.^15,16^

The stimulation of the left DLPFC was associated with pain inhibition in healthy individuals during acute pain.^17^ In contrast, perceived control of pain has been associated with activating the right DLPFC.^18^ Additionally, a neuroimaging study on depression linked hopelessness and poor judgment to prefrontal cortex left-right imbalance, with greater left-side hypoactivation.^19^ tDCS over the left DLPFC alleviates pain by reducing perfusion in the posterior insula and thalamus while modulating DLPFC–periaqueductal gray (PAG) interactions.^20^ These pathways integrate nociceptive input with cognitive and emotional processes, including patient expectations.^12^ Additionally, placebo effects are closely linked to enhanced DLPFC-PAG connectivity,^20^ while physical exercise activates motor area.^21,22^ A-tDCS on the left DLPFC with cathodal tDCS on the right DLPFC produced a top-down effect to improve pain catastrophizing and disability due to pain.^16^ Thus, bifrontal tDCS of the DLPFC is a promising therapy for improving interhemispheric balance.^23,24^ Additionally, meta-analyses have demonstrated greater pain relief when tDCS is combined with exercise compared with tDCS alone.^21,22^ However, the synergistic benefits of this combination require confirmation through rigorously controlled clinical studies, particularly to clarify the role of placebo effects alongside exercise and pain psychoeducation.

This trial aimed to evaluate whether multisession bifrontal home-based A-tDCS targeting the left DLPFC, combined with exercise and pain neuroscience education (PNE), is more effective than sham tDCS in reducing pain and disability, based on placebo-test responses (responders vs nonresponders).

## Methods

### Study Design and Participant Eligibility

The Hospital de Clínicas de Porto Alegre (HCPA) Research Ethics Committee approved this double-blind, sham-controlled randomized clinical trial. Participants provided written and oral consent. The trial protocol is provided in Supplement 1. We followed the Consolidated Standards of Reporting Trials (CONSORT) reporting guideline.^25^

The study involved right-handed, literate adult females between the ages of 18 and 65 years. These participants were recruited from the outpatient pain clinic Clinical Research Center of HCPA in Porto Alegre, Brazil, and through newspaper advertisements. All participants met the 2016 American College of Rheumatology criteria for fibromyalgia^26^ and reported a Numerical Pain Scale (NPS) score of 6 or higher on most days over the past 3 months. These patients agreed not to change their doses of antidepressants or anticonvulsants during the study. Exclusion criteria followed established guidelines for tDCS^27^ and uncompensated clinical conditions.

### Randomization and Blinding

**Figure 1.**
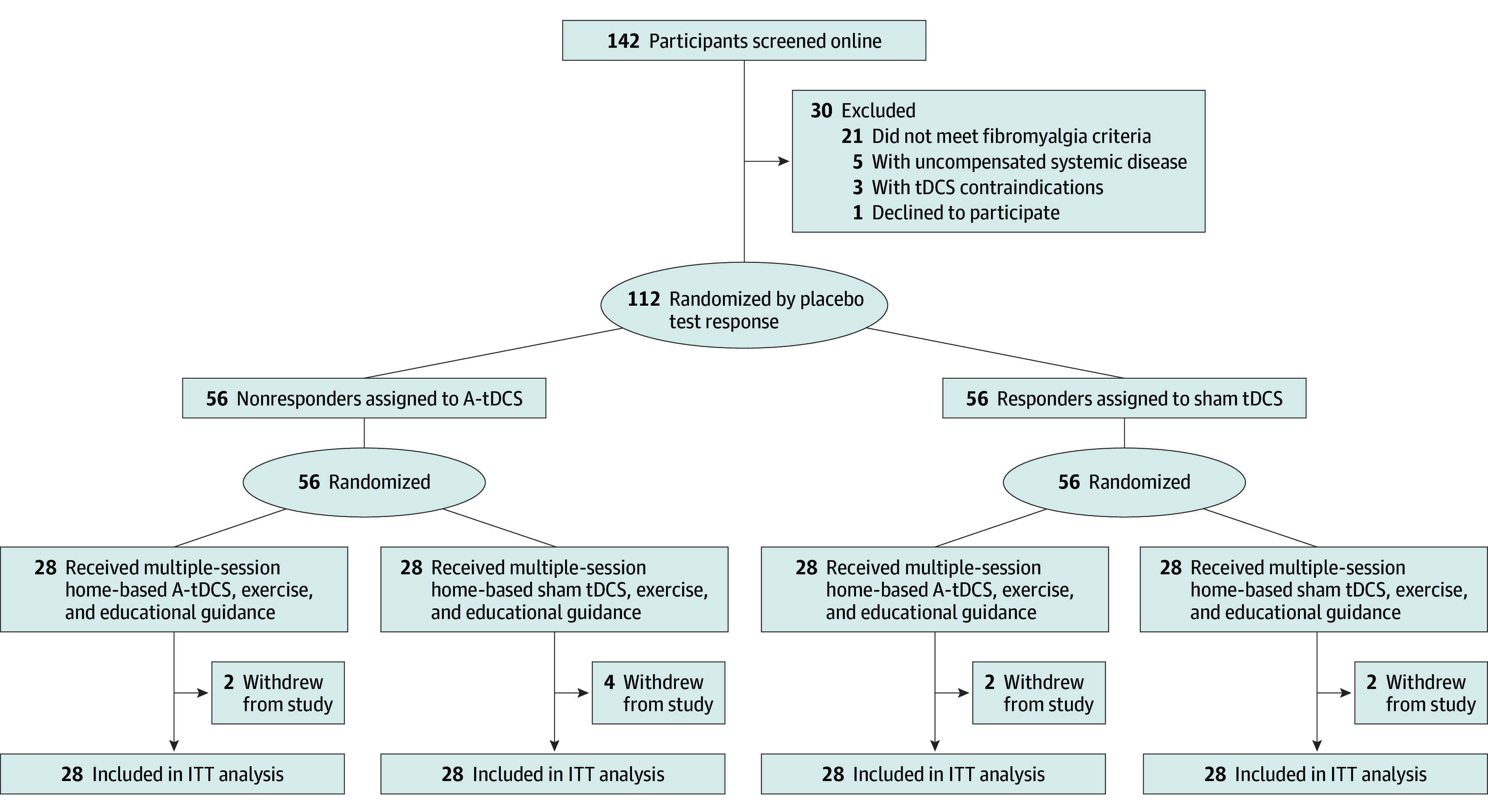
Study Flow Diagram A-tDCS indicates anodal transcranial direct current stimulation; ITT, intention to treat; tDCS, transcranial direct current stimulation.

Randomization was conducted between April 2022 and April 2024 using a randomization platform (Sealed Envelope; Sealed Envelope Ltd). Patients were assigned to each treatment group (A-tDCS or sham tDCS) and then stratified by placebo test response: responders (≥30% NPS score reduction) and nonresponders (<30% NPS score reduction). Patients were evenly split between the A-tDCS and sham tDCS groups and between responders and nonresponders (Figure 1). Two independent investigators (P.R.S.S. and D.P.D.S.) performed randomization in 10 blocks of 12 before recruitment. Randomization codes were sealed in brown envelopes and opened by the engineer (P.R.S.S.) programming the intervention devices.

The trial ensured rigorous blinding, with both participants and research staff being unaware of treatment allocation. An engineer programmed the tDCS device based on randomization, maintaining blinding throughout.

### Instruments and Assessments

The primary outcome was the change in Multidimensional Pain Interference Index (MPII) as measured by the Brief Pain Inventory (BPI; score range: 0-10, with 0 indicating no pain and 10 indicating worst pain) in 7 daily activities (general activity, walking, work, mood, enjoyment of life, relationships, and sleep) over 4 weeks of treatment and 3 months of follow-up. Overall interference was the mean NPS score across these 7 items.

Secondary outcomes were changes in pain-related disability, self-perception of improvement, quality of life, heat pain threshold (HPT), the endogenous pain modulatory system (EPMS), and treatment adherence. Disability due to pain was assessed through the Brazilian Profile of Chronic Pain: Screen (total score range: 0-93, with higher scores indicating greater disability or emotional distress), encompassing 3 domains: pain severity, disability in daily activities, and emotional burden.^28,29^ We assessed improvement impressions using the Patient Global Impression of Improvement Scale (posttreatment rating: 1 indicating very much worse to 7 indicating very much improved)^30^ and evaluated quality of life with the Fibromyalgia Impact Questionnaire (3 domains— function, overall impact, and symptoms—with maximum total score of 100)^31^ (Supplement 1). HPT was measured via Quantitative Sensory Testing (QST),^32^ and the EPMS was based on NPS score changes during the conditioned pain modulation (CPM) test.^33,34^ At time T0, the mean of 3 QST measurements at an NPS score of 6 of 10 was recorded. After 15 seconds of hand immersion in 0 to 1 °C water, QST at T0 determined NPS score (time T1). CPM test, calculated as the difference between NPS (T1) and NPS (T0), indicated EPMS function (negative score indicates proper function; ≥0 score indicates impaired function)^33,34^ (Supplement 1). Treatment adherence was tracked through session records (time, duration, intensity, and impedance), weekly walking logs (2-3 times for 20-30 minutes), and adverse events or safety via a standardized questionnaire. The presession exercise program was estimated at 20 minutes.

Demographic data and medical comorbidities were collected via a standardized questionnaire. Adverse effects of tDCS were evaluated using the Systematic Assessment for Treatment Evaluation questionnaire. The study used validated tools for the Brazilian population, including the Pittsburgh Sleep Quality Index (score range: 0-21, with higher scores indicating worse sleep quality),^35^ the Central Sensitization Inventory–Brazilian population (score range: 0-100, with higher scores indicating more severe symptoms),^36^ the Beck Depression Inventory II (score range: 0-13, indicating minimal depression; 14-19, indicating mild depression; 20-28, indicating moderate depression; 29-63, indicating severe depression),^37^ and the Pain Catastrophizing Scale (score range: 0-52, with higher scores indicating more pain catastrophizing).^38^

### Interventions

We used a home-based tDCS headset, developed and validated with HCPA’s Biomedical Engineering department and registered with Anvisa (Brazilian Health Regulatory Agency).^11,12,16,39^ The headset monitored contact impedance and session data, halting if impedance exceeded 1 mA for over 5 seconds or current fluctuated by more than 10%.

The intervention involved 20 sessions (2 mA, 20 minutes daily, 5 times per week for 4 weeks) with the anode over the left DLPFC (F3) and the cathode over the right DLPFC (F4). Sham tDCS (2 mA, 5 times per week for 4 weeks) included 30 seconds of stimulation at the start, then 10 minutes, and then 20 minutes, with a 20-second ramp-up and ramp-down. Electrodes (35 cm^2^) were moistened with saline and positioned using a neoprene cap. A biomedical engineer (P.R.S.S.) programmed the device for a minimum 16-hour interval between sessions and automatic control for both active and sham conditions (Supplement 1).

### PNE and Chronic Pain Management Protocol

The program includes motivational interviews, an exercise guide video (Video), and weekly WhatsApp messages (Supplement 1). It features 3 PNE videos (*Fibromyalgia as a Mind-Body Syndrome: Managing Symptoms and Improving Quality of Life*, *Central Sensitization: Loss of Inhibitory Capacity and Increased Pain Sensitivity*, and *Chronic Pain: The Effect of Active Participation in Treatment*) along with an exercise guide. The exercise program includes strengthening, flexibility, and aerobic activities (15-20 minutes daily before tDCS) plus aerobic walking (2-3 times per week based on tolerance).

**Video.** Daily Exercise Program Used Before Transcranial Direct Current Stimulation This video outlines the structured exercise program used in the trial, highlighting the importance of strengthening, flexibility, and aerobic exercises (15-20 minutes daily before transcranial direct current stimulation) as well as aerobic walking (2-3 times per week, based on individual tolerance).

### Statistical Analysis

The sample size was calculated to detect a 0.6-point difference in mean MPII values between A-tDCS vs sham tDCS at the final period (week 5) using a linear mixed-effects model (LMM). A total of 94 participants (47 per treatment group) provided 80% power at a 5% significance level. To account for 15% dropout, we increased the sample size to 112 (details are provided in Supplement 1). We analyzed categorical variables using the Fisher exact test; χ^2^ test; and continuous, unpaired, 2-tailed *t* test. The Shapiro-Wilk test assessed normality. For the primary and secondary outcomes (pain severity and disability), an LMM was used, incorporating fixed effects for treatment, placebo response, time, and their interaction along with a random intercept for patients. A generalized linear model (GLM) analyzed the treatment effect on quality of life and psychophysical measures. Treatment effects for both primary and secondary outcomes were adjusted for analgesic use and placebo response. The modified intention-to-treat (ITT) approach included participants who completed 50% or more of tDCS sessions, and missing MPII outcomes were imputed via regression using the treatment group as a predictor.^11,40^ The per-protocol analysis is presented in Supplement 1 and in the eMethods and eTable 1 in Supplement 2. Effect sizes were reported as Cohen *d*.

Two-tailed tests set at the 5% significance level were used, with Bonferroni correction for multiple comparisons. Analyses were conducted from July to December 2024 with SPSS software, version 22.0 (IBM).

## Results

A total of 142 patients were screened, of whom 30 were excluded, including 21 who did not meet diagnostic criteria and 8 who had chronic diseases or tDCS contraindications. As a result, 112 females (mean [SD] age, 49.04 [9.71] years) were randomized to either A-tDCS (n = 56 nonresponders) or sham tDCS (n = 56 responders) (Figure 1). Ten patients (4 from the A-tDCS group and 6 from the sham tDCS group) withdrew after completing 10 or more sessions mainly due to perceived ineffectiveness or discomfort. All withdrawals were included in the ITT analysis to reduce bias. Table 1 shows demographics and clinical characteristics, with no significant baseline differences between groups. Complete data were available for 51 patients (90.7%) in the sham tDCS group and 53 patients (93.57%) in the A-tDCS group.

**Table 1.** Sociodemographic and Clinical Characteristics by Treatment Group Classified as Placebo Test Responders vs Nonresponders

Characteristic	All participants, No. (%) (N = 112)	Respondents to placebo test, No. (%)			
		Nonresponders (n = 56)		Responders (n = 56)	
		Sham tDCS (n = 28)	A-tDCS (n = 28)	Sham tDCS (n = 28)	A-tDCS (n = 28)
Age, mean (SD), y	49.04 (9.71)	47.05 (9.11)	49.32 (7.67)	48.24 (13.77)	51.58 (8.32)
Formal education, mean (SD), y	11.63 (3.58)	12.39 (3.50)	12.32 (3.49)	11.71 (4.38)	10.11 (2.96)
Working condition, yes					
Working	68 (60.7)	18 (64.3)	19 (67.9)	14 (50.0)	17 (60.7)
Unemployed	24 (21.4)	4 (14.3)	7 (25.0)	5 (17.9)	8 (28.6)
Health license	10 (8.9)	5 (17.9)	1 (3.6)	3 (10.7)	1 (3.6)
Retired	10 (8.9)	1 (3.6)	1 (3.6)	6 (21.4)	2 (7.1)
Smoking, yes	21 (18.8)	4 (14.3)	5 (18.9)	8 (28.6)	4 (14.3)
Alcohol use, yes	24 (21.4)	5 (17.9)	7 (25.0)	3 (10.7)	8 (28.6)
Diagnosis of psychiatric disorder, yes^a^	60 (53.6)	13 (46.4)	12 (42.9)	18 (64.3)	17 (60.7)
Panic disorder	21 (18.8)	4 (14.3)	5 (17.9)	7 (25.0)	5 (17.9)
Major depressive disorder	60 (53.6)	13 (46.4)	12 (42.9)	18 (64.3)	17 (60.7)
Anxiety disorder	17 (15.2)	4 (14.3)	5 (17.9)	5 (17.9)	3 (10.7)
Analgesic use, yes^b^	69 (61.6)	14 (50.0)	19 (67.9)	18 (64.3)	18 (64.3)
Opioid analgesic^b^	21 (18.8)	6 (21.4)	6 (21.4)	6 (21.4)	3 (10.7)
Codeine	5 (4.5)	1 (3.6)	1 (3.6)	3 (10.7)	0
Tramadol	20 (17.9)	6 (21.4)	5 (17.9)	6 (21.4)	3 (10.7)
Nonopioid analgesic^b^	68 (60.7)	17 (60.7)	16 (57.1)	18 (64.3)	18 (64.3)
Dipyrone	17 (15.2)	7 (25.0)	3 (10.7)	4 (14.3)	3 (10.7)
Acetaminophen	30 (26.8)	7 (25.0)	10 (35.7)	7 (25.0)	6 (21.4)
NSAID	21 (18.8)	8 (28.6)	6 (21.4)	4 (14.3)	3 (10.7)
Muscle relaxants, yes	19 (17.0)	7 (25.0)	3 (10.7)	5 (17.9)	4 (14.3)
SSRI, yes	34 (30.4)	8 (28.6)	7 (25.0)	9 (32.1)	10 (35.7)
Duloxetine, yes	38 (33.9)	9 (32.1)	11 (39.3)	9 (32.1)	9 (32.1)
Tricyclic antidepressant, yes	24 (21.4)	6 (21.4)	7 (25.0)	7 (25.0)	4 (14.3)
Pregabaline, yes	33 (29.5)	7 (25.0)	8 (28.6)	10 (35.7)	8 (28.6)
Gabapentin, yes	14 (12.5)	5 (17.9)	5 (17.9)	2 (7.1)	2 (7.1)
2016 ACR fibromyalgia criteria					
Global score, mean (SD)^c^	19.54 (3.44)	19.57 (3.44)	20.32 (3.22)	20.71 (3.91)	19.71 (3.53)
WPI, mean (SD)^d^	10.78 (2.36)	10.78 (2.36)	11.53 (2.04)	11.21 (2.66)	10.96 (2.86)
SSS score, mean (SD)^e^	8.78 (1.49)	8.78 (1.49)	8.68 (1.72)	9.50 (1.81)	8.75 (1.85)
CSI-BP score, mean (SD)^f^	65.88 (15.12)	62.32 (14.09)	68.71 (15.43)	69.67 (16.25)	62.82 (14.74)
PSQI, mean (SD)^g^	16.67 (5.21)	17.10 (5.23)	16.64 (4.73)	16.85 (6.15)	16.10 (4.73)
PCS score, mean (SD)^h^	36.74 (9.62)	36.28 (8.49)	36.32(9.62)	37.03 (11.60)	37.32 (8.77)
Pain on VAS, mean (SD)^i^	7.77 (1.18)	7.86 (1.11)	7.79 (1.10)	7.75 (1.14)	7.68 (1.36)
No. of valid tDCS sessions, mean (SD)	18.50 (2.34)	18.34 (2.69)	18.20 (2.88)	18.95 (1.70)	18.50 (2.10)
Regular walking 2-3 times per wk, mean (SD), yes	69 (17.25)	16 (57.14)	15 (53.57)	18 (64.28)	20 (71.42)

Abbreviations: ACR, American College of Rheumatology; A-tDCS, anodal transcranial direct current stimulation; CSI-BP, Central Sensitization Inventory–Brazilian population; NSAID, nonsteroidal anti-inflammatory drug; PCS, Pain Catastrophizing Scale; PSQI, Pittsburgh Sleep Quality Index; SSRI, selective serotonin reuptake inhibitor; SSS, Severity Symptoms Scale; tDCS, transcranial direct current stimulation; VAS, visual analog scale; WPI, Widespread Pain Index.

^a^ Patients could have none or more than 1 psychiatric diagnosis.

^b^ Some patients were taking more than 1 type of drug.

^c^ Global score range: 0-31, with higher scores indicating more severe symptoms.

^d^ WPI score range: 0-19, with higher scores indicating more severe symptoms.

^e^ SSS score range: 0-12, with higher scores indicating more severe symptoms.

^f^ CSI-BP score range: 0-100, with higher scores indicating severe symptoms.

^g^ PSQI score range: 0-21, with higher scores indicating worse sleep quality.

^h^ PCS score range: 0-52, with higher scores indicating more pain catastrophizing.

^i^ VAS score range: 0-10, with higher scores indicating more severe symptoms.

### Primary Outcome

**Figure 2.**
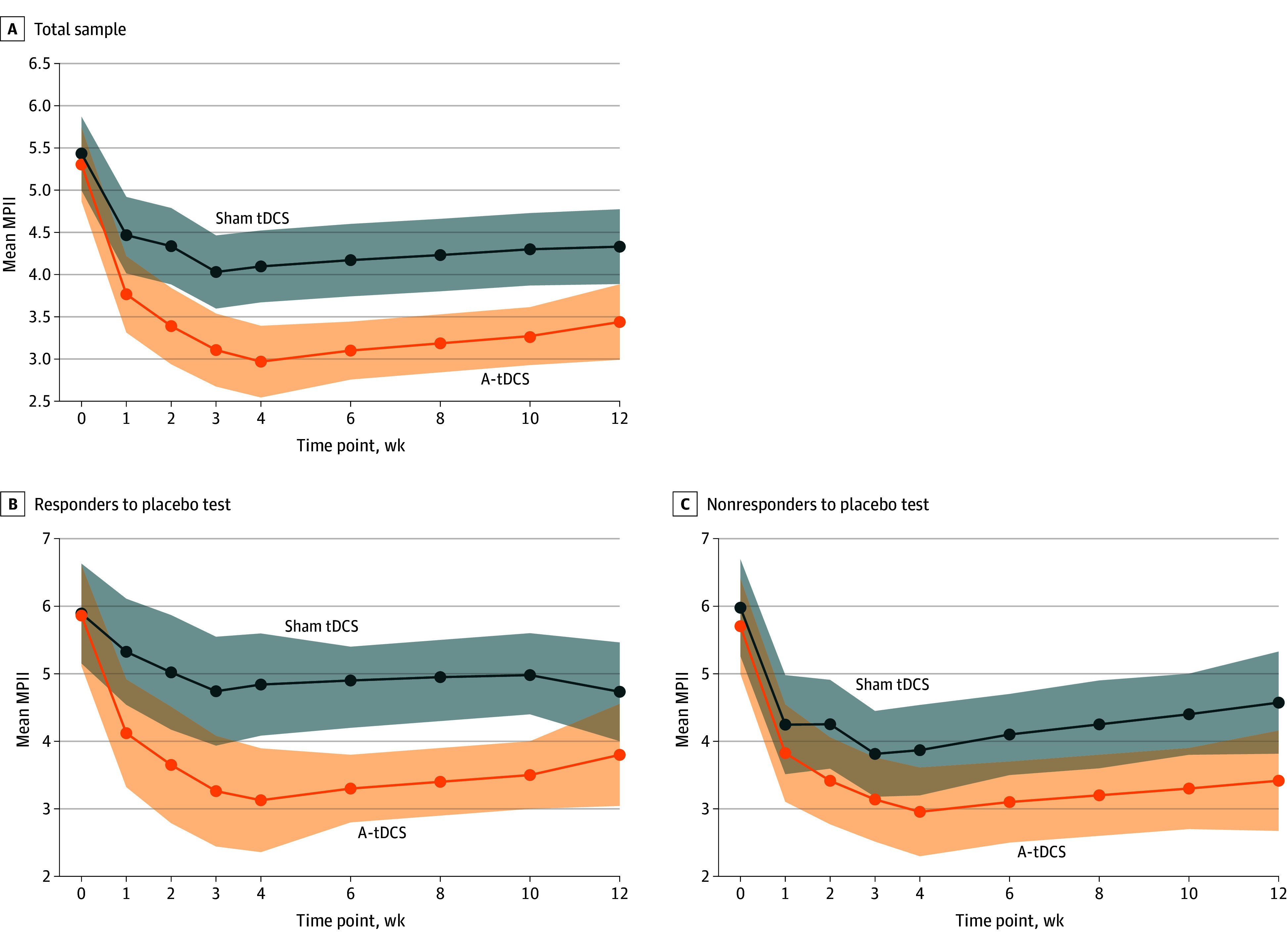
Change in Multidimensional Pain Interference Index (MPII) Over Time Linear mixed-effects models assessed the effect of treatment—anodal transcranial direct current stimulation (A-tDCS) vs sham transcranial direct current stimulation (tDCS)—combined with exercise and pain neuroscience education on MPII (as measured by the 7-item Brief Pain Inventory), adjusted for analgesic use. Solid lines represent mean scores at each time point (weeks 0-12), and shaded areas represent 95% CIs. Overlapping CIs indicate no differences between treatments.

LMM analysis (ITT) showed a significant MPII reduction for A-tDCS vs sham tDCS (38.76% [95% CI, −41.90% to −30.92%] vs 16.08% [95% CI, −21.42% to −10.41%]; mean difference [MD], 22.68% [95% CI, 12.79%-40.00%]; Cohen *d* = 0.73) across treatment and follow-up (*F* = 31.45; *P* = .003), with no treatment-by-time interaction (*F* = 1.48; *P* = .19) (Table 2, Figure 2). A placebo effect was observed (*F* = 7.78; *P* = .003) with no interaction with treatment (β = −0.47; 95% CI, −0.60 to 1.46; *P* = .11). In placebo responders, MPII was reduced by 34.21% (95% CI, −46.88% to −28.29%) for A-tDCS vs 18.13% (95% CI, −24.90% to 3.34%) for sham tDCS (MD, 24.23%; 95% CI, 15.80%-32.67%). Among placebo nonresponders, MPII decreases were 35.49% (95% CI, −41.21% to −29.53%) for A-tDCS vs 25.96% (95% CI, −34.31% to −20.42%) for sham tDCS (MD, 9.52%; 95% CI, 2.79%-19.78%).

**Table 2.** Primary Outcome: Change in Multidimensional Pain Interference Index by Placebo Test Response

Outcome	Sham tDCS		A-tDCS	
	Mean (SD)	Change from baseline (95% CI), %	Mean (SD)	Change from baseline (95% CI), %
MPII as measured by BPI (n = 56)				
Baseline	5.96 (1.87)	NA	5.78 (1.61)	NA
Week 1	4.79 (1.86)	−14.72 (−25.71 to −3.72)	4.02 (1.87)	−32.06 (−36.78 to −15.92)
Week 2	4.63 (1.94)	−22.51 (−25.71 to −3.72)	3.53 (1.88)	−39.81 (−45.26 to −28.10)
Week 3	4.46 (1.79)	−19.33 (−33.337 to −11.35)	3.29 (1.67)	−42.45 (−47.99 to −26.91)
Week 4 (end of treatment)	4.61 (1.78)	−12.45 (−28.74 to 2.96)	3.03 (2.23)	−48.86 (−59.66 to −37.67)
3-mo follow-up	4.70 (2.02)	−11.40 (−22.50 to 3.08)	3.71 (1.38)	−30.67 (−44.07 to −22.08)
Cumulative relative change	NA	−16.08 (−21.42 to −10.41)	NA	−38.76 (−41.90 to −30.92)
Responders to placebo test (n = 28)				
Baseline	6.03 (2.61)	NA	6.05 (1.90)	NA
Week 1	5.24 (2.76)	−13.10 (−19.31 to 15.41)	4.17 (2.11)	−31.07 (−42.69 to −7.41)
Week 2	4.93 (3.04)	−18.24 (−29.12 to −1.41)	3.59 (1.76)	−40.66 (−51.03 to −23.90)
Week 3	4.65 (2.84)	−22.88 (−33.50 to 10.20)	3.20 (1.63)	−47.10 (−66.18 to −21.74)
Week 4 (end of treatment)	5.04 (2.69)	−16.41 (−21.93 to 38.34)	3.07 (1.41)	−49.26 (−81.29 to −19.95)
3-mo follow-up	4.82 (2.33)	−20.06 (−24.96 to 8.50)	3.98 (1.39)	−30.31 (−47.88 to −12.75)
Cumulative relative change	NA	−18.13 (−24.90 to 3.34)	NA	−34.21 (−46.88 to −28.29)
Nonresponders to placebo test (n = 28)				
Baseline	6.03 (1.78)	NA	5.86 (1.89)	NA
Week 1	4.86 (1.71)	−19.40 (−40.99 to −16.92)	3.75 (2.69)	−36.00 (−39.29 to −15.66)
Week 2	4.17 (1.67)	−30.84 (−40.22 to −18.92)	3.33 (2.31)	−43.17 (−45.76 to −24.65)
Week 3	4.10 (1.76)	−32.00 (−41.14 to −14.22)	3.24 (2.21)	−44.70 (−51.18 to −24.76)
Week 4 (end of treatment)	4.35 (2.06)	−27.86 (−44.23 to −24.38)	3.48 (2.39)	−40.61 (−58.54 to −39.05)
3-mo follow-up	4.84 (2.42)	−19.73 (−31.13 to 78.25)	3.78 (2.91)	−27.30 (−46.97 to −7.81)
Cumulative relative change	NA	−25.96 (−34.31 to −20.42)	NA	−35.49 (−41.21 to −29.53)

Abbreviations: A-tDCS, anodal transcranial direct current stimulation; BPI, Brief Pain Inventory; MPII, Multidimensional Pain Interference Index; NA, not applicable; tDCS, transcranial direct current stimulation.

Higher pain interference in daily activities was associated with greater analgesic use (β = 0.18; 95% CI, 0.01 to 0.34; *P* = .04). Estimated marginal means (EMM [SD]) for MPII were 3.86 (1.19) for A-tDCS vs 4.78 (1.27) for sham tDCS, with an MD (pooled SD) of −0.90 (1.57). The effect size was moderate (Cohen *d* = 0.57). Per-protocol analysis is provided in eMethods in Supplement 2.

The analysis based on placebo test responses (responders vs nonresponders) showed a larger effect size of A-tDCS in responders. EMM (SD) for MPII was 4.01 (1.14) for A-tDCS vs 5.12 (1.13) for sham tDCS (*F* = 24.00; *P* < .001), with an MD (pooled SD) of 1.13 (1.14) and a large effect size (Cohen *d* = 0.86). In nonresponders, EMM (SD) was 3.76 (1.04) for A-tDCS vs 4.45 (1.12) for sham tDCS (*F* = 14.69; *P* = .008), with an MD (pooled SD) of 0.69 (1.08) and a moderate effect size (Cohen *d* = 0.63).

### Secondary Outcomes

GMM analysis revealed that A-tDCS vs sham tDCS reduced BPI pain severity over 24 hours during treatment and follow-up (*F* = 8.37; *P* = .002). A placebo effect (*F* = 8.98; *P* = .009) and placebo-treatment interaction (β = −0.88; 95% CI, −1.61 to −0.15; *P* = .01) were observed. EMM (SD) for BPI mean pain score was 4.10 (1.26) for A-tDCS vs 4.64 (1.28) for sham tDCS, with a moderate effect size (Cohen *d* = −0.43) and an MD (pooled SD) of 0.54 (1.27).

A-tDCS did not significantly reduce the BPI worst pain score (*F* = 0.56; *P* = .45). A placebo effect (*F* = 4.84; *P* = .02) and placebo-treatment interaction (β = −0.20; 95% CI, −0.70 to −0.02; *P* = .04) were observed. Higher BPI scores (mean and worst pain) were associated with an increase in analgesic use of 0.23 (95% CI, 0.13-0.33) points, equivalent to a mean (range) increase of 23.3% (13%-33%).

Details on relative changes in main effects on pain severity and Brazilian Profile of Chronic Pain: Screen over time are provided in eTables 2 and 3 in Supplement 2). For example, we observed that A-tDCS, compared with sham tDCS, reduced pain by 12.77 (95% CI, 4.60-20.94) points.

### Effects of Treatment 

LMM analysis showed that A-tDCS vs sham tDCS reduced pain-related disability (*F* = 5.96; *P* = .01). EMM (SD) was 62.52 (9.95) for A-tDCS vs 66.98 (12.16) for sham tDCS, with a moderate effect size (Cohen *d* = −0.4) and an MD (pooled SD) of −4.46 (11.01). No placebo effect or analgesic use influence was observed (eTable 2 in Supplement 2).

Thirty-five participants (62.5%) in the A-tDCS group and 21 participants (37.5%) in the sham tDCS group achieved 50% or more improvement, with a 40% relative risk reduction (0.60; 95% CI, 0.39-0.91) in pain interference in daily functioning. According to the Patient Global Impression of Improvement Scale, 40 participants (71.1%) who received A-tDCS vs 28 participants (50.9%) who received sham tDCS reported 30% or more improvement, while 48 (84.9%) vs 31 (55.0%), respectively, felt much or moderately better (eTable 4 in Supplement 2).

The GLM showed that A-tDCS, compared with sham tDCS, improved quality of life (MD, −15.22 [18.78] vs −9.43 [12.42]; mean [SD] score after treatment: 56.89 [19.41] vs 64.36 [17.52]), HPT (MD, 0.43 [1.96] vs −0.65 [1.86]; mean [SD] score after treatment: 37.48 [1.79] vs 37.72 [1.82]), and inhibitory system efficiency (CPM test) (MD, 0.21 [2.33] vs 0.35 [2.53]; mean [SD] score after treatment: −1.75 [1.70] vs −1.32 [1.91]) (Table 3). Additionally, A-tDCS significantly reduced fibromyalgia’s impact on quality of life, with a greater effect among placebo test responders vs nonresponders (β = 6.31; 95% CI, 0.59-12.04) and a moderate effect size (Cohen *d* = 0.41). No placebo effect was observed in the CPM test or HPT.

**Table 3.** Secondary Outcomes: Change in Quality of Life and Psychophysical Pain Measures by Treatment Group

Outcome	Mean (SD) before treatment	Mean (SD) after treatment	Mean difference (SD)	β (SEM) [95% CI]	Wald χ^2^	*df*	*P* value	Cohen *d*
FIQ score^a^								
A-tDCS (n = 56)	72.42 (14.0)	56.89 (19.41)	−15.22 (18.78)	5.74 (1.16) [0.15 to 4.41]	3.89	1	.04	0.38
Sham tDCS (n = 56)	73.97 (14.97)	64.36 (17.52)	−9.43 (12.42)	1 [Reference]	NA	NA	NA	NA
Responder to placebo	NA	NA	NA	6.31 (2.93) [0.59 to 12.04]	4.67	1	.03	0.41
Heat pain threshold, °C^a^								
A-tDCS (n = 56)	37.05 (1.54)	37.48 (1.79)	0.43 (1.96)	1.09 (.35) [0.38 to 1.79]	9.25	1	<.001	0.60
Sham tDCS (n = 56)	38.39 (2.01)	37.72 (1.82)	−0.65 (1.86)	1 [Reference]	NA	NA	NA	NA
Responder to placebo	NA	NA	NA	0.13 (0.36) [−.57 to 0.84]	0.83	1	.71	NC^b^
NPS score during CPM test^c^								
A-tDCS (n = 56)	−1.96 (2.18)	−1.75 (1.70)	0.21 (2.33)	0.93 (0.42) [1.79 to 4.89]	4.89	1	.02	0.43
Sham tDCS (n = 56)	−1.75 (1.96)	−1.32 (1.91)	0.35 (2.53)	1 [Reference]	NA	NA	NA	NA
Responder to placebo	NA	NA	NA	0.43 (0.42) [−0.38 to 1.26]	1.78	1	.29	NC^b^

Abbreviations: A-tDCS, anodal transcranial direct current stimulation; CPM, conditioned pain modulation; *df*, degree of freedom; FIQ, Fibromyalgia Impact Questionnaire; NA, not applicable; NC, not calculated; NPS, Numerical Pain Scale; SEM, standard error of the mean; tDCS, transcranial direct current stimulation.

^a^ Comparisons between treatment groups used the mean difference value by generalized linear models, with the outcomes as continuous variables.

^b^ Not calculated because there was no significant difference between treatment groups.

^c^ Outcomes as dichotomous variables in a binary logistic regression analysis. In the CPM test, participants were classified as nonresponders when scores were equal to or higher than 0 and as responders when scores were lower than 0. All analyses were followed by Bonferroni correction to check for differences between groups and to correct for multiple comparisons.

### Assessment of Adherence, Adverse Events, and Safety

Adherence to the tDCS protocol (Table 1) was based on completed sessions. Although 20 sessions were required, 12 patients (5 in the A-tDCS group and 7 in the sham tDCS group) completed fewer than 15 sessions. The A-tDCS group completed 1013 of 1120 sessions (91.2%) and the sham tDCS group completed 1028 of 1120 sessions (91.8%), with overall adherence of 91.2% (2041 of 2240 sessions). Among participants in the sham tDCS vs A-tDCS group, adherence was 60.0% (12 of 20 days) vs 63.0% (13 of 20 days) for walking and 92.5% (18.5 of 20 days) vs 90.2% (18 of 20 days) for pretreatment exercise at least 3 days per week.

The most common adverse effects for A-tDCS vs sham tDCS were pain in the stimulation area (40 [71.5%] for both), tingling (28 [50.0%] vs 31 [54.2%]), and burning (35 [62.1%] vs 25 [48.1%]). Headache was more frequent in patients in the sham tDCS vs A-tDCS group (23 [40.6%] vs 16 [29.9%]), whereas sleepiness (23 [41.1%] vs 18 [32.3%]) and mood changes (33 [59.8%] vs 28 [49.5%]) were more common in the A-tDCS vs sham tDCS group. Most symptoms were similar across groups and were classified as mild to moderate, even in those who discontinued due to burning (eTable 5 in Supplement 2).

## Discussion

In line with our hypothesis, 20 sessions of home-based A-tDCS over 4 weeks bifrontal A-tDCS on the left DLPFC combined with exercise and PNE reduced pain severity, disability, and pain interference assessed by the MPII, particularly in participants prone to a placebo response. These findings align with previous results of A-tDCS research in clinical settings^13,41,42^ and support the home-based tDCS protocol for fibromyalgia.^11,12,14,16,43^ The treatment benefits persisted for 3 months but gradually decreased, likely due to neuroplastic adaptation.^44^ From a neurobiological perspective, bifrontal A-tDCS over the left DLPFC may regulate pain by modulating key brain regions involved in pain processing and emotional regulation. Left DLPFC stimulation enhances cognitive control over pain-related emotions,^16,43,45,46^ while right DLPFC stimulation reduces stress-related hyperactivity.^42^ This finding aligns with evidence that A-tDCS decreases perfusion in the posterior insula and thalamus and modulates DLPFC-PAG interactions, reducing pain perception.^47^

The moderate effect size in reducing pain severity and improving disability in individuals receiving A-tDCS aligns with meta-analyses showing a similar effect size when combining noninvasive brain stimulation and exercise for chronic pain.^22^ While exercise type and intensity can affect outcomes, this combination has been explored in few studies.^44,48,49^ Our trial protocol combined unsupervised at-home exercise with PNE, making it more translatable to routine care. This approach enhances scalability and patient empowerment, offering a realistic intervention for clinical settings. Despite similar adherence rates for walking and pre-tDCS exercise, walking adherence was lower than expected but aligned with fibromyalgia-related aerobic difficulties.^50,51^ However, the trial’s parallel design limited our ability to isolate the effects of each intervention or explore potential synergies. Since all participants received the same exercise and PNE instructions to minimize bias, the differences observed in participants who received A-tDCS are likely due to the stimulation itself.

The high response rate to A-tDCS (71.1%) resulted in more than 30% reduction in pain interference, with 84.9% reporting much or moderate improvement. This high rate of positive perception in the A-tDCS group aligns with existing evidence on placebo effects in clinical outcomes.^52,53^ The placebo effect influenced pain intensity. While it interacted with pain severity measures, the placebo susceptibility did not affect pain interference as measured by BPI nor affected psychophysical measures (ie, CPM test and HPT). The placebo effect may alter pain perception but is insufficient for lasting improvements in pain interference, which require active neuromodulation and behavioral adaptations. This finding aligns with the lack of placebo effects on psychophysical measures, reflecting their distinct mechanisms.^54^ Pain intensity ratings are subjective, shaped by expectation, emotions, and cognitive modulation, making them more susceptible to placebo effects.^54^ In contrast, psychophysical measures primarily reflect spinal and brainstem–mediated pain processing, which remains less influenced by expectation-driven cortical mechanisms.^55^

To our knowledge, this trial has been the largest to combine A-tDCS with exercise and PNE, potentially minimizing placebo responses, compared with clinic-based trials. Placebo responders in the sham tDCS group showed a slight reduction in pain and disability and a pattern of returning to baseline level at follow-up, likely driven by expectations and regression to the mean.^56,57^ The A-tDCS effect was greatest in responders, which aligns with evidence that individuals more susceptible to placebo tend to respond better to treatment. In contrast, among placebo nonresponders, the sustained improvement suggests a more genuine A-tDCS effect.^58,59^ These effects should be interpreted within the trial protocol, which combined home-based bifrontal tDCS, exercise, and PNE. While this approach may enhance prefrontal cortex-driven neuroplasticity, improving pain perception and coping,^60,61^ home-based tDCS may have reduced the placebo response by eliminating routine clinic visits. The high adherence to home-based tDCS (≥90%) supports its clinical application in fibromyalgia treatment.

### Limitations

This trial has several limitations. First, it included only females to address sex-related differences in pain processing^62^ and EPMS inhibition.^63^ Second, while loss to follow-up could introduce bias, it was random and balanced across groups, with all participants completing at least 10 sessions, which is considered to be sufficient for chronic pain improvement.^64^ However, the per-protocol analysis indicated this loss to follow-up had no significant effect on results. Third, sham tDCS was associated with more adverse effects, possibly due to heightened vigilance, negative expectations,^65^ and issues with electrode placement. Fourth, the adverse effects of A-tDCS were generally mild to moderate, consistent with a previous study.^11,43,66,67^ However, there was a lack of explicit assessment of participant blinding at the end of the trial, which could have provided additional validation of the sham control effectiveness. Future studies should address assessment of blinding to clarify the relationship between expectations, placebo effects, and treatment efficacy.

## Conclusions

This sham-controlled randomized clinical trial showed that bifrontal A-tDCS, with A-tDCS on the left DLPFC, combined with exercise and PNE effectively improved disability due to pain. The findings of this study support fibromyalgia management and enhance understanding of tDCS-related placebo effects.

## Notes

Supplement 1.
Trial Protocol

Supplement 2.
eMethods.
**eTable 1.** Multidimensional Pain Interference Index: Per Protocol Analysis of Interventions **eTable 2.** Pain Severity (Average and Worst Pain) and Disability Due to Pain (B-PCP Total Score) **eTable 3.** LMM Analysis of the Intervention Groups (a-tDCS vs s-tDCS) on BPI Pain Severity and B-PCP Total Score, Including Their Interactions With Time and Placebo-Test Response (n = 112) **eTable 4.** Patient Impression of Improvement **eTable 5.** Side Effects Presented as Percentage, and the Incidence or Severity of Side Effects Classified as Absence, Mild, Moderate, and Severe (n = 112)

Supplement 3.
Video Daily Exercise Program Used Before Transcranial Direct Current

Supplement 4.
Data Sharing Statement
