# Error in the Byline

**DOI:** 10.1001/jamanetworkopen.2025.26572

**Published:** 2025-07-17

In the Original Investigation titled “Home-Based Transcranial Direct Current Stimulation vs Placebo for Fibromyalgia: A Randomized Clinical Trial,”^1^ which was published on June 6, 2025, the spelling of Dr Pacheco-Barrios’ surname was incorrect. This article has been corrected.
