# Error in the Byline

**DOI:** 10.1001/jamanetworkopen.2025.40518

**Published:** 2025-10-02

In the Original Investigation titled “Home-Based Transcranial Direct Current Stimulation vs Placebo for Fibromyalgia: A Randomized Clinical Trial,”^1^ which was published on June 6, 2025, Mr França de Souza’s surname was listed incorrectly. This article has been corrected.
