# Supplementary material for: Home-Based Transcranial Direct Current Stimulation vs Placebo for Fibromyalgia: A Randomized Clinical Trial
Source: JAMA Netw Open. 2025 Jun 6;8(6):e2514262. doi: 10.1001/jamanetworkopen.2025.14262 (PMC12144624; doi:10.1001/jamanetworkopen.2025.14262)
Supplement: Supplement 1. — Trial Protocol [file jamanetwopen-e2514262-s001.pdf]

Index

Title: Effect of Home-based Transcranial Direct Current Stimulation vs Placebo Stimulation for Fibromyalgia: FIBRO-HB-tDCS Randomized Clinical Trial

c. Principal Investigator 2

a. Theme 2

b. Objective 2

c. Social relevance 2

d. Objectives 4

2. Home-Based tDCS Device 4

3. Electrodes position and tDCS stimulation protocol 6

e. Study setting: 6

f. Study population 6

f.1. Recruitment 6

f.2. Inclusion Criteria 7

f.3. Exclusion Criteria 7

f.4.. Sample size estimation 7

g. Randomization 9

h. Blinding 10

i. Ethical guarantees for study participants: 10

j. Methods 10

Table 1. Schedule of assessment. 17

Schedule of activities 17

Statistical Analysis 18

Risks and Benefits of the Study: 17

Study Discontinuation Criteria 19

Study Results 19

p. Dissemination of Results 19

o. REFERENCES 20

Project: Effect of Home-based, Self-applied, Transcranial Direct Current Stimulation vs Placebo Stimulation for Fibromyalgia: FIBRO-HB-tDCS Randomized Clinical Trial

Study Protocol (original translated version submitted to the Ethics Committee of the Hospital de Clínicas de Porto Alegre, Federal University of Rio Grande do Sul, Porto Alegre, Brazil, on October 16th, 2021).

Principal Investigator:

Wolnei Caumo, M.D., Ph.D.

Research Assistants:

- Barbara Regina Franca, Psychologist, Master's Degree.
- Roman Orzechowski, M.D., Master's Degree.

a. Theme

The subject of this study is the multiple-session home-based self-applied anodal transcranial direct current stimulation (A-tDCS) to the left dorsolateral prefrontal cortex (l-DLPFC), combined with exercise (E) and pain neuro-education guidance (PNE), influence pain and disability due to pain, considering the propensity to respond to placebo-test effect.

b. Objective

To compare the effects of multisession (a-tDCS) targeting the PFC, combined with exercise (E) and pain education (PEN), versus sham tDCS (s-tDCS) on pain severity, daily activity interference (BPI), and pain-related disability (Br-PCP). Secondary outcomes included quality of life, heat pain threshold (HPT), changes in the Numerical Pain Scale (NPS 0-10) during the conditioned pain modulation test (CPM-test), Patient Global Impression of Improvement (PGI-I), and treatment adherence.

c. Social relevance:

c.1. Fibromyalgia affects 2% to 5.4% of the population, causing chronic pain, fatigue, sleep issues, and cognitive-emotional symptoms [1]. Medication adherence is challenging since 72.5% of patients do not follow their prescribed treatments [2], and 67% of duloxetine users show poor adherence [3]. Duloxetine, amitriptyline, gabapentin, and pregabalin provide at least 50% pain relief in only 10–15% [4]. So, guidelines recommend non-pharmacological interventions [5–7]. The benefits of delivering transcranial direct current stimulation (tDCS) treatment at home are highlighted, emphasizing reduced burdens, improved compliance, and enhanced access for diverse populations. We presented a validated home-based (HB)-tDCS protocol, offering clear guidelines for safety, user education, standardization, and quality control [8]. The protocol includes details on the HB-tDCS device characteristics, electrode positions, stimulation protocols, and user training. Notably, it emphasizes user selection considerations, the importance of well-defined protocols permitting patient-scheduled sessions, and adherence monitoring. The document also outlines a structured study protocol, incorporating baseline assessments, training sessions, and approaches to monitoring protocol compliance. In conclusion, the

perspectives on tDCS with a structured protocol at home might contribute significantly to advancing neuromodulation practices considering the susceptibility to placebo effect.

c.2. tDCS is a noninvasive technique that uses scalp electrodes to modulate neuronal excitability and neuroplasticity with low electrical currents (0.5–2 mA)[9,10]. Stimulating the left dorsolateral prefrontal cortex (l-DLPFC) can alleviate depressive symptoms, improve cognitive function, and reduce pain catastrophizing and disability [11–13]. In contrast, stimulation of the primary motor cortex (M1) is particularly effective at reducing pain severity [11]. However, tDCS requires daily application, which can create logistical challenges when done in a clinical setting, including travel burdens and constraints due to clinic hours and staff availability [14]. HB-tDCS, validated for remote use, addresses this challenge and has shown benefits in FM symptom management across studies involving 20 to 60 sessions [15,16]. The effectiveness of anodal-(a)-tDCS is influenced by the stimulation site, duration, and intensity, underscoring the importance of combining therapies to guide neuroplasticity [17].

c.3. Aerobic exercise can boost neuroplasticity and enhance tDCS outcomes [18]. Meta-analyses show greater pain relief when combined with exercise compared to tDCS alone [19]. However, their efficacy needs to be confirmed in rigorously controlled clinical studies considering the role of placebo, especially when combined with exercise and pain education, needs further understanding. Several studies have explored transcranial direct current stimulation (tDCS) in various clinical settings. They have demonstrated effectiveness (Level A) in treating depression.

Several studies have explored (tDCS in various clinical settings, demonstrating its effectiveness (Level A) in treating depression. It is also likely effective (Level B) for conditions such as neuropathic pain, fibromyalgia, migraines, Parkinson's disease (both motor and cognitive symptoms), stroke (motor), epilepsy, schizophrenia, alcohol and drug addiction, and obesity [11,20,21(p6),22,23]. However, the widespread adoption of tDCS faces challenges due to the necessity of multiple sessions and the costs associated with patients traveling to medical center, which also affects the healthcare system.

To overcome this barrier, home-based tDCS devices have been developed, providing greater convenience for patients and reducing the need for frequent trips to medical centers[24]. Despite this advancement, clear guidance for practical therapeutic applications of tDCS remains a challenge [25]. Establishing a standardized protocol for tDCS is crucial to enhance treatment compliance and improve accessibility, especially for individuals in remote areas or those facing physical, cognitive, or chronic health challenges requiring long-term tDCS use [26].

While many tDCS approaches involve interactions between supervisors and users, the devices must be programmable in a personalized manner and include safety features to prevent protocol changes without supervision. At the same time, patients must have access to support when necessary. Our experience in randomized controlled trials supports the positive outcomes of tDCS [13,15,16,27].The success of tDCS depends on carefully selecting users based on their physical and cognitive capacities to operate the device safely. A comprehensive protocol for tDCS is essential, encompassing safety, education, standardization, ethics, quality control, community engagement, accessibility, and regulatory compliance.

d. Objectives

This is clinical trial aims to compare the efficacy of Multiple-session home-based self-applied anodal tDCS (a-tDCS) targeting the left dorsolateral prefrontal cortex (l-DLPFC) the long-term effects of combining exercise (E) and pain neuroeducation guidance (PNE), based on placebo-test responses (responders vs. non-responders) in the following outcomes:

Primary outcome: Brief Pain Inventory (BPI), which allows pain assessment from a multidimensional perspective (pain intensity and interference in general activities, mood, mobility, work, personal relationships, sleep and enjoyment of life, etc.) (primary outcome).

Secondary outcomes: To assess the effectiveness of multiple-session home-based self-applied anodal tDCS (a-tDCS) targeting the left dorsolateral prefrontal cortex (l-DLPFC) and the long-term effects of combining exercise (E) and pain education guidance (PNE), considering susceptibility to the placebo effect, on (1) the impact of pain on quality of life; (2) heat pain threshold; and (3) the function of the descending pain modulatory system. Additionally, the study aims to identify predictors of response to the placebo effect through a hierarchical model with an analytical structure defined a priori. This model will consider the hierarchical relationships between potential predictors, such as disability due to pain, catastrophizing, depressive symptoms, psychiatric diagnoses, level of central sensitization, baseline endorphin serum levels, current medications, and other relevant factors.

2. Home-Based tDCS Device

The device for HB-tDCS is registered with the Brazilian National Health Surveillance Agency (ANVISA) under number Nº80079190028. This device was developed and validated for use at home, as demonstrated by its previous use in different trials conducted by our group [13,27,28]. The device monitors contact impedance at a sampling rate of 1 mA and interrupts the session if the impedance exceeds a predetermined value of 1 mA for an interval of 5 seconds or if the electric current undergoes an alteration greater than 10%. The equipment records the time and duration of use, as well as the session time, which allows for adherence monitoring. Since July 2020, when this protocol was in progress, it has been commercialized by Mendes and Barbosa Produtos Médicos Ltda Quark Medical (Brazil). The tDCS equipment for home use is presented in Figure panel (**1, 2, 3, 4, 5 and 6**).

The Home-Based tDCS device represents a significant advancement in at-home neuromodulation therapies, offering patients a user-friendly and effective option for managing chronic pain conditions. In collaboration with the Biomedical Engineering department at HCPA in Porto Alegre, Brazil, the Laboratory of Pain and Neuromodulation developed and validated this home-based tDCS device, ensuring secure usage while monitoring and recording session quality and duration [8,28]. The device is registered with the ANVISA under number Nº80079190028 and has been specifically designed for home use, as demonstrated by its application in various trials conducted by our research group [13,27,28]. The device features a monitoring system for contact impedance at a sampling rate of 1 mA, which automatically interrupts the session if impedance exceeds a predetermined threshold of 1 mA for 5 seconds or if there is a fluctuation in electric current greater than 10%. Additionally, it records usage time, duration of sessions, and overall compliance, facilitating adherence monitoring. Since July 2020, this protocol has been commercialized by Mendes and Barbosa Produtos Médicos Ltda Quark Medical (Brazil). The tDCS equipment for home use is illustrated in figure panels (1**, 2, 3, 4, 5, and 6)**.

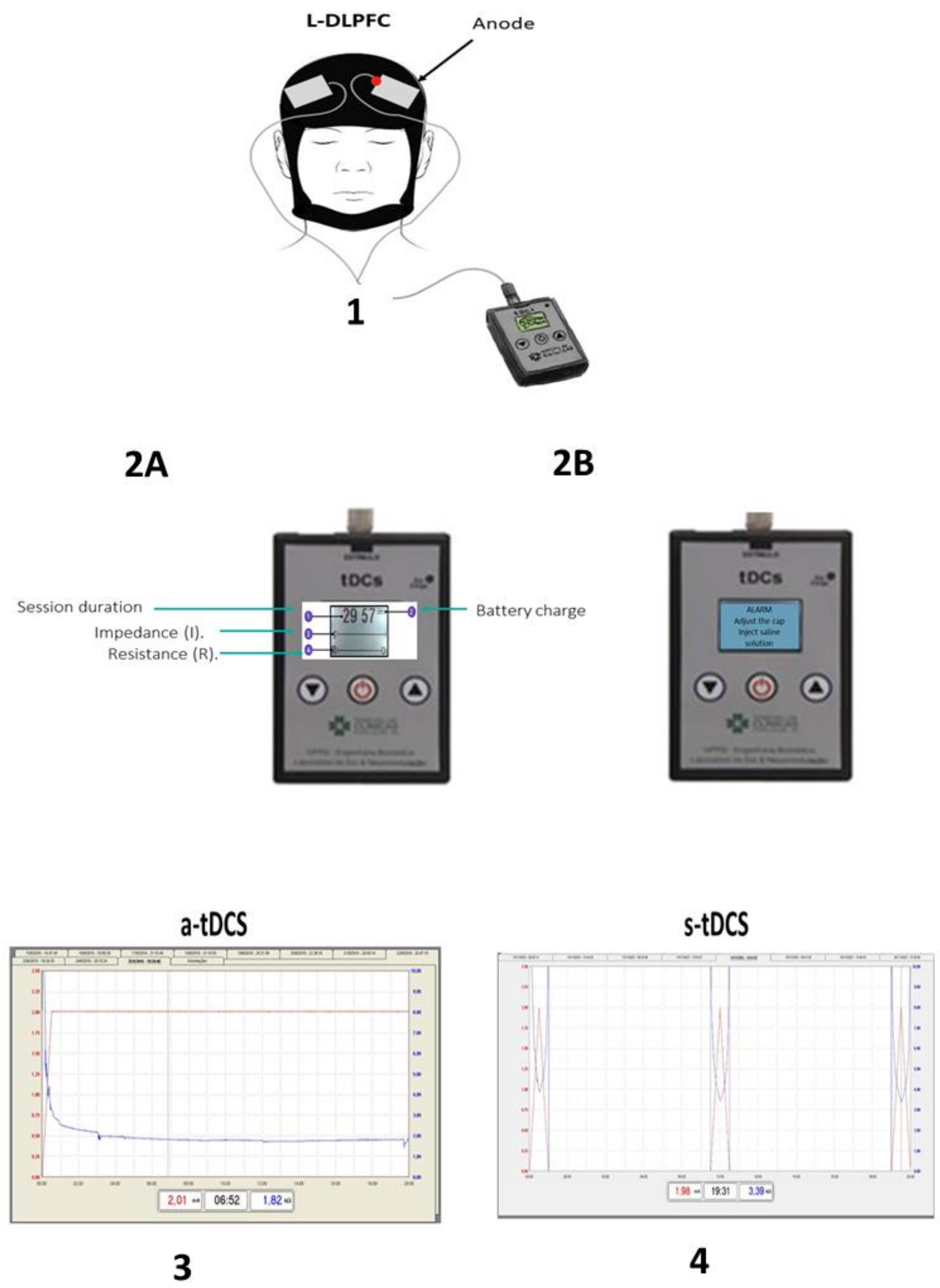

Figure panel (**1, 2A and 2B, 3, 4**).

(1) Schematic drawing of the electrode**.** Flexible Vinyl material, conductive rubber, and vegetal sponge on the Neoprene cap of 4 mm thickness manufactured by Biomedical Engineering Department of Hospital de Clínicas de Porto Alegre, Porto Alegre, Brazil, with anodal transcranial Direct Current Stimulation (a-tDCS) placed over left DLPFC (F3) and cathode over right DLPFC (F3). **Device Features: (2A)** High resistance. **(2B)** Alarm warning: adjust the cap and inject extra saline. **(3)** Typical curves of current intensity versus contact impedance during a tDCS. **(4)** Typical curves of current intensity versus contact impedance during 30 seconds in s-tDCS.

3. Electrodes position and HB-tDCS stimulation protocol

This study protocol used scalp electrodes positioned according to the 10-20 system for EEG, with the anode at F3 and the cathode at F4 and the current applied for 20 minutes at 2 mA (see Figure 1). The study included both a-tDCS and sham conditions, with the device programmed to offer 30 seconds of stimulation at the beginning, after 10 minutes, and after 20 minutes during the

sham conditions. To ensure sham conditions, the same montage was used for both a-tDCS and s-tDCS. Both active and s-tDCS had a ramp-up time of 20 seconds for the current to reach 2 mA, followed by a ramp-down time of 20 seconds. This protocol was designed to mimic active tDCS but without delivering sustained stimulation. The electrodes are 35cm2 and coated with a vegetable sponge moistened with saline solution administered by two silicone cannulas coupled to the electrode. The positioning of the electrodes in the stimulation sites was facilitated using a neoprene elastic cap available in varied sizes for proper adjustment to the patients' heads. To ensure the blinding of participants regarding the intervention they received the device was programmed to automatically turn on and off at each of the specified time points for both active and s-tDCS conditions. A biomedical engineer programed the device to provide a predetermined number of stimulation sessions, with a minimum interval of 16 hours between consecutive sessions. This protocol was designed to ensure that participants did not receive tDCS sessions extra than the programmed in the protocol, which could potentially lead to adverse effects or interfere with the outcomes of the study. This approach was adopted to ensure that participants were unaware of whether they were receiving active or sham stimulation at any given time during the study.

The study's protocol included visits to the center for baseline assessment, training on how to use the device, and assessment at treatment end. The participants receive instructions on how to self-apply with the tDCS, choose a quiet time in their daily schedule to apply the treatment session, and record adverse effects. The treatment protocol for tDCS at home involved several steps to ensure proper use and adherence to the protocol.

e. Study setting: Center for Clinical Research at Hospital de Clínicas de Porto Alegre, affiliated with the Federal University of Rio Grande do Sul (UFRGS), Porto Alegre, Brazil.

f. Study population

Recruitment

Patients were selected from the Pain Services at HCPA, Basic Health Units, and the HCPA Physiatry Service. Informational brochures outlining the inclusion criteria were distributed to the Basic Health Units, allowing primary care physicians to refer patients. Community volunteers were also able to contact the research team via email. The community was informed about the study through announcements made by the Communication Service of the Hospital de Clínicas de Porto Alegre. Candidates were contacted by phone, and those who met the inclusion criteria were invited for a medical evaluation, clinical history collection, and a detailed description of their symptoms to confirm the diagnosis. Advertisements were also posted online (e.g., Facebook.com, craigslist.org, etc.). During the initial contact (typically a phone call), the researcher conducted a screening questionnaire that included confidential medical information (such as a history of drug and alcohol abuse as exclusion criteria). The diagnosis was reviewed or established by a trained physician. Data obtained from the screening were stored in a file managed by the responsible researcher.

To prevent potential losses, the initial phone contact was detailed. Subjects were informed about the time commitment required for the procedures involved. Researchers made themselves available for any further clarifications, either by phone or in person.

Inclusion Criteria

Women aged 18 to 75 years; right-handed, literate, with a confirmed diagnosis of fibromyalgia (FM) according to the criteria of the American College of Rheumatology (2010-2016). These criteria required assessment using the widespread pain index (WPI) and the symptom severity score (SSS). Specifically, participants needed either a WPI score of at least seven, or a score between 4 and 6 accompanied by an SSS of 9 or higher or widespread pain in four out of five regions along with specific symptoms such as abdominal pain, depression, and headache within the last six months. In general, a combined WPI and SSS score of 13 or more was necessary as per the ACR-2016 standards. Moreover, participants were required to report a minimum score of 6 on the Numerical Pain Scale (NPS), which ranges from 0 to 10, for most days over the previous three months. They also had to agree not to change their doses of antidepressant and anticonvulsant medications during the study.

Exclusion Criteria

Residents outside the Greater Porto Alegre area; pregnancy; decompensated systemic diseases; chronic inflammatory rheumatological diseases; uncompensated hypothyroidism; personal history of cancer currently or in treatment; history of alcohol or drug abuse in the last six months; decompensated psychiatric disorders with defined suicide risk. Contraindications for tDCS: metallic implants in the brain; implanted medical devices in the brain; pacemakers; cochlear implants; neurological pathologies; history of traumatic brain injury or neurosurgery.

f.4. Sample size estimation

The primary outcome of this study, pain-related function, is measured using the 7-item Brief Pain Inventory (BPI) interference scale. This scale serves as a Multidimensional Pain Interference Index (MPII), providing a comprehensive view of how pain affects various dimensions of an individual's life, including emotional well-being, social interactions, and daily activities. Focusing solely on pain severity may fail to reflect the full impact of pain on a person's overall quality of life. The secondary outcome, pain intensity, is evaluated using the 2-item BPI severity scale. Both BPI scales range from 0 to 10, with higher scores representing worse function or intensity. Understanding the Minimal Clinically Important Differences (MCID) changes in pain interference scores is vital for clinicians to determine whether changes are significant enough to indicate meaningful improvements or declines in quality of life, rather than just being statistically significant [29]. According to previous studies in chronic pain populations, an MCID of approximately 0.7 points for both the BPI interference and severity scales has been identified [30]. However, consensus guidelines have established a 1-point difference as the MCID for both scales, while a 30% reduction from baseline is also recognized as a significant indicator of moderate improvement [31]. In examining the effects of E-PNE with a-tDCS and s-tDCS groups at the final time point (moment 5), which occurs three months post-treatment, a more conservative MCID of 0.6 points was used for estimating the sample size. This cautious approach aims to ensure a thorough evaluation of the study's power to assess the treatment effects. The sample size estimation was performed using the PSS *Health tool 32. https://hcpa-unidade-bioestatistica.shinyapps.io/PSS_Health/).* To detect a minimum difference of 0.6 points between the means of the Multidimensional Pain Interference Index (MPII) for the a-tDCS group and the s-tDCS group at the final time point (moment 5) in a repeated measures analysis, a sample size of 94 participants (47 per group) was calculated. To account for a potential dropout rate of 15%, the sample size was increased to 112 participants. This calculation was based on an

assumed 80% power, a 5% significance level, and relevant parameters for the treatment group.

Standard deviation of 1 unit

- Retention rates of 99%, 98%, 96%, and 94% at moments 2, 3, 4, and 5, respectively
- Autoregressive correlation matrix for MPII by BPI of 0.4 for the a-tDCS group

For the s-tDCS :

- Standard deviation of 1 unit
- Retention rates of 99%, 98%, 96%, and 94% at moments 2, 3, 4, and 5
- Autoregressive correlation parameter of 0.2.

R code:

```
Ra <- matrix(data = c(1, 0.4, 0.16, 0.064, 0.0256, 0.4, 1, 0.4, 0.16, 0.064, 0.16, 0.4, 1, 0.4, 0.16, 0.064, 0.16, 0.4, 1, 0.4, 0.0256, 0.064, 0.16, 0.4, 1), nrow = 5, byrow = TRUE)
Rb <- matrix(data = c(1, 0.2, 0.04, 0.008, 0.0016, 0.2, 1, 0.2, 0.04, 0.008, 0.04, 0.2, 1, 0.2, 0.04, 0.008, 0.04, 0.2, 1, 0.2, 0.0016, 0.008, 0.04, 0.2, 1), nrow = 5, byrow = TRUE)
longpower::power.mmrm(Ra = Ra, ra = c(100, 99, 98, 96, 94)/100, sigmaa = 1, Rb = Rb, rb = c(100, 99, 98, 96, 94)/100, sigmab = 1, delta = 0.6, lambda = 1, sig.level = 5/100, power = 80/100)
```

Minimum detectable difference	Standard deviation Treatment	Standard deviation Control	Significance level (%)	Power (%)	Ratio of sample size	Sample size Treatment	Sample size Control	Sample size
0.6	1	1	5	80	1	47	47	94
1	1	1	5	80	1	17	17	34
1.4	1	1	5	80	1	9	9	18
1.8	1	1	5	80	1	6	6	12
2.2	1	1	5	80	1	4	4	8
2.6	1	1	5	80	1	3	3	6
0.6	1.2	1	5	80	1	56	56	112
1	1.2	1	5	80	1	21	21	42
1.4	1.2	1	5	80	1	11	11	22
1.8	1.2	1	5	80	1	7	7	14
2.2	1.2	1	5	80	1	5	5	10
2.6	1.2	1	5	80	1	3	3	6
0.6	1.5	1	5	80	1	73	73	146
1	1.5	1	5	80	1	27	27	54
1.4	1.5	1	5	80	1	14	14	28
1.8	1.5	1	5	80	1	9	9	18
2.2	1.5	1	5	80	1	6	6	12
2.6	1.5	1	5	80	1	4	4	8

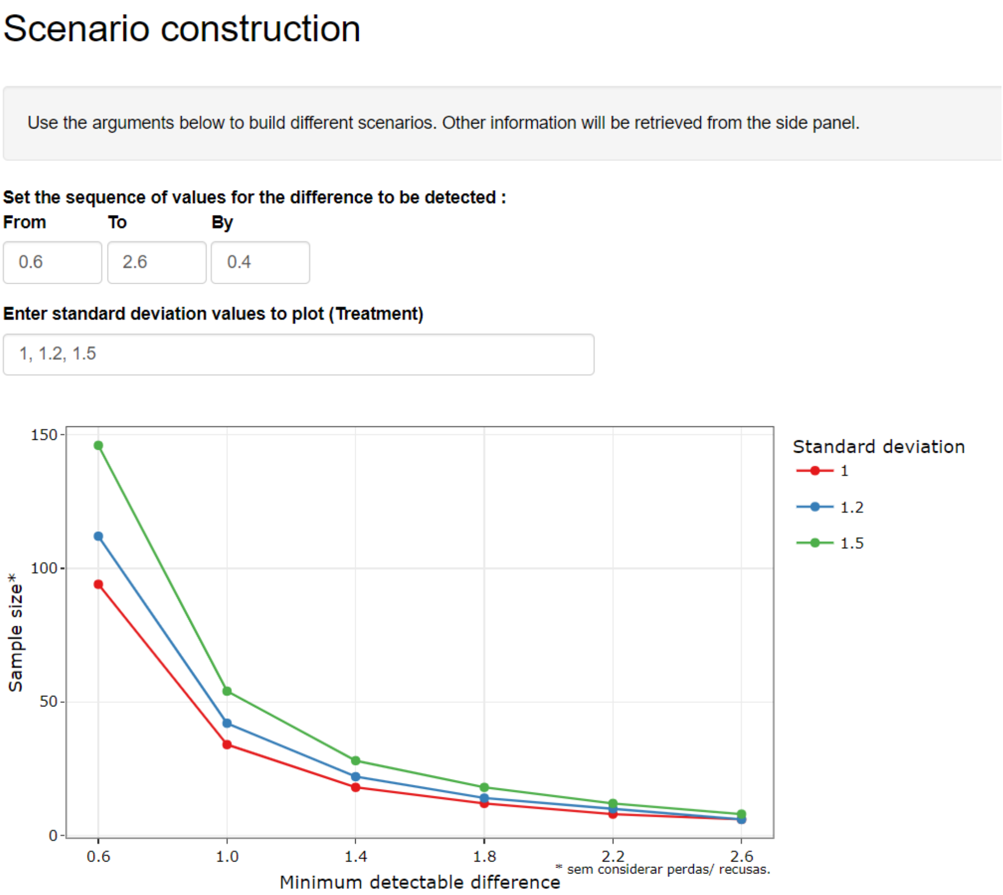

g. Randomization

Using the website www.sealedenvelope.com, random numbers were employed to fairly distribute 112 patients into two groups: those who would receive anodal transcranial direct current stimulation (a-tDCS) or sham transcranial direct current stimulation (s-tDCS) over the dorsolateral prefrontal cortex (DLPFC). The allocation ratio was based on susceptibility to the placebo response, determined by the reduction in the NPS (0-10) from pre- to after one session of s-tDCS. Participants were classified as responders if their NPS (0-10) decreased by 30% or more or non-responders if it was less than 30%. This fair process resulted in a 1:1:1:1 allocation ratio, with 56 patients receiving a-tDCS (28 responders and 28 non-responders) and 56 patients receiving s-tDCS (28 responders and 28 non-responders). To prevent predicting the next patient's allocation, randomization was performed in 10 blocks of twelve. Prior to the recruitment phase, two investigators who were not involved in the patients' assessments performed the randomization. The randomized numbers were placed in sealed, brown envelopes with the patient's entry sequence number written outside. The engineer responsible for programming the intervention device opened the envelope only after the patient had provided informed consent.

h. Blinding

The study enforced rigorous protocols to maintain blinding among participants, research staff, and investigators regarding treatment allocation. The researcher responsible for receiving the tDCS device was unaware of whether it was programmed for active or sham stimulation, as an engineer handled this programming determined by randomization.

i. Ethical guarantees for study participants:

The research was conducted by a team of trained physicians and psychologists based at the University Hospital of the Federal University of Rio Grande do Sul (UFRGS), Porto Alegre, Brazil. Patient triage will be performed by this team at the Hospital de Clínicas de Porto Alegre (HCPA), and clinical information will be collected with strict confidentiality. Participants will be thoroughly informed about all scientific and ethical aspects of the research in accessible language, tailored to their biopsychosocial context. They were enrolled in the study after providing written informed consent, with each participant retaining a copy of the signed document.

j. Methods

The study protocol is outlined in the figure of timeline the study (**figure 2**), showing the assessments conducted at each stage.

The tDCS device used in the study was developed in collaboration with the Biomedical Engineering department of the Hospital de Clínicas de Porto Alegre (HCPA), Brazil. It is registered on the Brazilian National Health Surveillance Agency (ANVISA) under registration number Nº80079190028. This device, designed for home use, has demonstrated its effectiveness in several trials [8,13,15,16].

Key features of the device include the ability to monitor contact impedance and automatically stop the session if impedance exceeds 1 mA for more than 5 seconds or if there is an electric current fluctuation greater than 10%. The device also logs the time and duration of each session, ensuring patient adherence. Since July 2020, it has been commercially available through Quark Medical (Brazil).

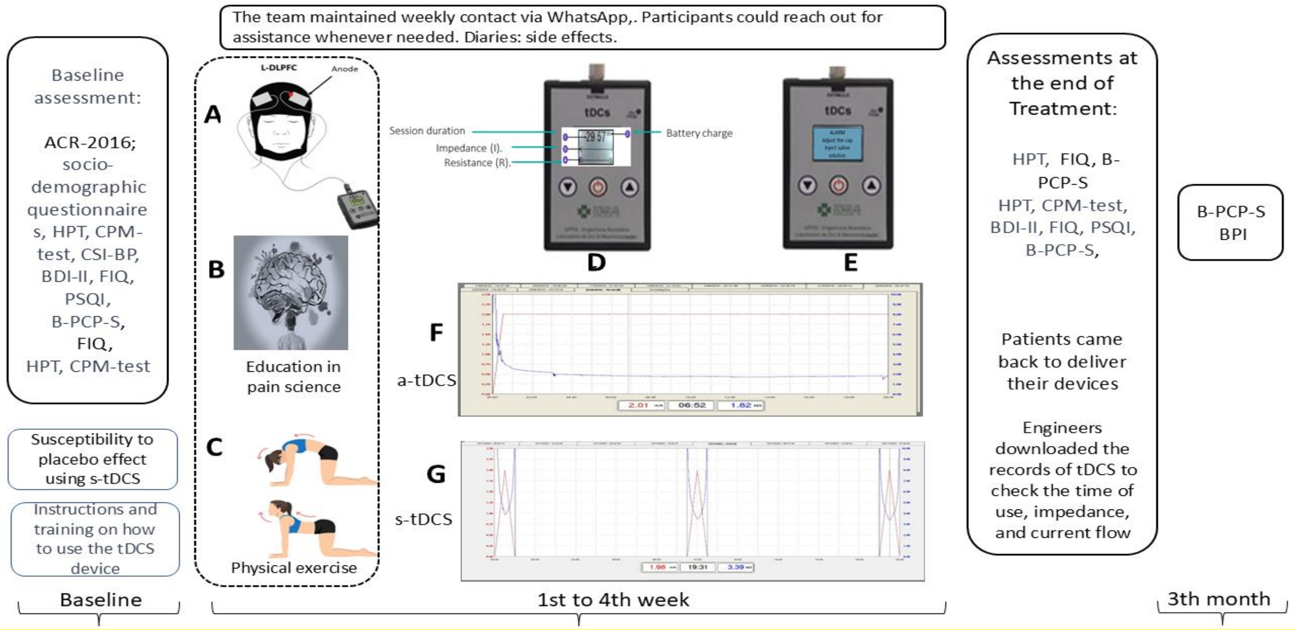

Figure 2. Timeline the study.
Protocol Components: The stimulation sites for the study include anodal tDCS with the anode placed over the left dorsolateral prefrontal cortex (DLPFC) (F3) and the cathode over the right DLPFC (F4). Additional components of the protocol involve pain education and physical exercise. The device features a high resistance and alarm system to adjust the cap and add saline, as well as monitoring curves of current intensity versus impedance for both active (a-tDCS) and sham (s-tDCS) stimulation.

Assessments: Participants completed a baseline questionnaire covering clinical and socio-demographic characteristics, analgesic use, heat pain threshold (HPT), and pain pressure threshold (PPT) assessed via quantitative sensory testing (QST).

Instruments: The study utilized several validated instruments, including the Fibromyalgia Impact Questionnaire (FIQ), Central Sensitization Inventory (CSI-BP), Beck Depression Inventory (BDI-II), Brazilian Pain Catastrophizing Scale (BP-PCS), Pittsburgh Sleep Quality Index (PSQI), and the Brazilian Portuguese version of the Profile of Chronic Pain: Screen (B-PCP).

The study's protocol consisted of a structured sequence:

Baseline Assessment: This began at the center, where participants were initially assessed.

Training Session (Visit 1): Volunteers received comprehensive training on using the tDCS device correctly. They were provided with instructions for self-administration at home. The cap size and electrode positions were determined, and participants were given a link to a video guide for self-administration *(https://youtu.be/3Wtji4esOGE).* They were also encouraged to contact the research team if needed.

Visit 2: Occurred four weeks after starting the tDCS protocol. Participants returned to the center for a treatment-end assessment and returned the device. Throughout the protocol, participants were required to use the device appropriately and keep records of any adverse effects experienced.

Cap Size and Electrode Positioning

The process of determining cap size and electrode positions involved several steps:

(i) Measuring the participant's head circumference to select the appropriate cap size (small, medium, or large).

(ii) Participants wore the cap while the researcher identified electrode positions following the 10-20 EEG system.

(iii) Electrode positioning on the cap was based on randomization, with scenarios such as anode at F3 and cathode at F4 (see Figure 1).

(iv) Electrodes were inserted into vegetable sponges and secured in the cap following the 10-20 EEG system.

(v) To avoid electrode placement errors, the anode was marked red, and the cathode was marked black.

(vi) Detailed instructions for self-administration of the tDCS device at home can be found here: *https://www.jove.com/video/57614/home-based-transcranial-direct-current-stimulation-device-development.*

Training and Self-Application of tDCS at Home

The training and instructions for participants to self-administer tDCS at home included the following steps:

(i) Participants received initial training on equipment usage and skin irritation identification.

(ii) The first treatment session was supervised with detailed instructions provided through a step-by-step video guide.

(iii) Participants were instructed to prepare the stimulation area by exposing it in front of a mirror.

(iv) The skin under the electrodes was cleaned with alcohol to remove creams, dirt, or grease.
(v) Participants were guided to wear the cap, positioning the seam between their eyebrows.
(vi) Approximately 6 ml of saline was added to the syringes connected to the sponges.

HB-tDCS Sessions and Compliance Monitoring

To ensure compliance with protocol during HB-tDCS sessions, we implemented several procedures:

(i) Participants were advised to choose a quiet and suitable time in their daily schedule for their treatment session.
(ii) The initial home-based session was conducted under remote supervision, allowing participants to seek assistance from the research team if needed.
(iii) A weekly contact through WhatsApp to track participants' progress and ensure they adhered to the treatment protocol.
(iv) Participants were encouraged to contact the research team for assistance.
(v) Participants were instructed to promptly document any adverse effects in a diary following each tDCS home session.

To verify the adherence to the following protocol, a biomedical engineer reviewed the records to confirm the session time, duration, current intensity, and contact impedance. The average impedance was employed to assess the quality of the delivered current. The impedance should range between 8 and 4 kΩ at the beginning of stimulation, with the target during treatment between 3 and 2 kΩ. For a valid 20-minute a-DCS session, impedance levels should be between 3 and 2 kΩ for at least half the time in the case of a duration of 10 min. The literature supports the choice of a 10-minute duration by demonstrating its effectiveness in promoting neuroplasticity, with studies by Nitsche (2000) [20] and Bikson (2016) [33] adding to this understanding.

Exercises and Pain Science Education (PNE)

One of the consequences of chronic pain is the fear of experiencing it and the avoidant behavior that immobilizes patients. This behavior hinders patients' active participation in treatment, which motivates the search for strategies that prioritize behavioral change to educate patients about the importance of their active involvement in alleviating symptoms and rehabilitating. Therefore, treatment includes strategies to modify coping capacity. Pain neuroscience education (PNE) programs aim to improve adherence and reduce fear and anxiety. PNE uses pain experience and associated consequences to clarify the neurophysiological-endocrine-immune-motor changes produced by pain to enhance the understanding of chronic pain as a disease, not a symptom. These programs aim to reframe individual perceptions of pain and make it less threatening**, empowering patients to take control of their pain management** [34]. According to data from a systematic review with meta-analysis, which included qualitative and quantitative studies, PNE in chronic pain produces a clinically relevant effect in reducing kinesiophobia and pain catastrophizing [35].

PNE is presented through motivational interviewing to motivate participants to perform the proposed exercises and actively confront persistent pain and related symptoms. This model considers the role of beliefs and understanding

of one's disease (identity, cause, consequence, control, and duration) [36,37]. A physical therapist supervises the implementation of videos on neuroeducation and exercise that are sent and made available via a mobile app. The orientation to perform the exercises is reinforced weekly through WhatsApp messages. The neuroeducation about pain was in a face-to-face evaluation on the day they received training to use the tDCS equipment, with questions directed at fear, cognitive distortion, and avoidance. This approach through motivational interviewing aligns with scientific evidence supporting the importance of the patient telling their own story regarding their pain experience, thus enabling a reconceptualization of chronic pain as a disease [35]. The four videos address the following aspects:

Video 1: Fibromyalgia as a mind-body syndrome – managing symptoms and improving quality of life despite the lack of a cure *(https://youtu.be/37ULMvGDk3s).*Fibromyalgia is a chronic pain syndrome marked by widespread pain, fatigue, sleep disturbances, and cognitive issues, primarily affecting women and significantly reducing quality of life. It stems from altered brain pain regulation, heightening pain sensitivity. Though incurable, a multimodal treatment approach can help manage symptoms: (i) Medications alleviate pain and improve sleep but do not eliminate symptoms. (ii) Physical exercise may reduce pain, enhances mobility, and builds strength. (iii) Complementary therapies, like tDCS show promise as adjunctive treatments. Therefore, a comprehensive care plan empowers patients to better manage symptoms and enhance their quality of life.

- **Video 2: Fibromyalgia and central sensitization** – how the loss of inhibitory capacity makes the brain more sensitive to pain *(https://youtu.be/MpSNGLTs5Wk).* The video aim to give insights for patients about fibromyalgia as a complex condition marked by widespread pain, fatigue, sleep disturbances, and mood changes, due to central sensitization, where the brain becomes overly sensitive to pain. The brain loses its ability to inhibit pain, intensifying discomfort. Managing symptoms effectively involves strategies like physical exercise and tDCS, which stimulates the brain to reduce pain. Combining these treatments with professional guidance is essential for better symptom control and improving quality of life.
- **Video 3: Fibromyalgia as a chronic pain condition characterized by dysfunction of the central system** – stressors exacerbate symptoms, but focusing on quality of life and active participation in treatment enhances outcomes *(https://youtu.be/oJOpurJRJQ4).* Effective management requires a multimodal approach, including: (i) Regular Exercise improves physical function and reduces pain. (ii) Education helps identify and manage triggers. (iii) tDCS therapy enhances treatment effectiveness.
- Video 4: **Exercise program –** strengthening, flexibility, and aerobic activities to be performed before each tDCS session *(https://youtu.be/l95-5YwgFeE).* This video provides a Complete Guide with detailed instructions on exercises

to complement tDCS sessions for fibromyalgia. The recommended program includes strengthening exercises for the upper and lower limbs and axial regions using body weight, functional movements, and alternating limb exercises. Key exercises include squats (sitting and standing from a chair) and bilateral plantar flexions. Flexibility is addressed through stretching targeting the upper and lower limbs, lumbar, and cervical spine. The program includes two blocks of five exercises, with at least two sets, adjusted based on fatigue and pain levels. Exercises are performed daily before tDCS sessions for 15–20 minutes, complemented by aerobic walking 2–3 times per week based on tolerance.

- **Exercise Program for Study Participants**
    1. Seated, elbow flexion, palms up.
    2. Seated, knee extension, one at a time.
    3. Seated, shoulder flexion, sagittal plane, bilateral.
    4. Standing, hip flexion 90°, one at a time.
    5. Standing, hip abduction, one at a time.
    6. Standing, shoulder abduction, coronal plane, bilateral.
    7. Standing, bilateral plantar flexion.
    8. Sit and stand from a chair (minimum of 43 cm from the ground).
    9. Cervical region mobility – flexion and extension, rotations, and inclinations.
    10. Trunk mobility – flexion and extension, rotations, and inclinations.
    11. Deep diaphragmatic breathing technique.

Instruments and Assessments

The tools used to measure psychological and clinical outcomes were validated in the Brazilian population. The schedule of assessment is presented in Table 1.

Primary Outcome

i. Pain interference was assessed using a multidimensional pain score from the Brief Pain Inventory (BPI), which evaluates seven daily activities: general activity, walking, work, mood, enjoyment of life, relationships, and sleep. BPI pain interference scores range from 0 to 10, with 0 indicating no interference and 10 indicating complete interference. Overall pain interference was calculated as the mean of the seven items. The assessment was conducted over a 4-week treatment period and at three months of follow-up [38].

Secondary Outcomes:

ii. The Brief Pain Inventory (BPI) assesses pain at its "worst," "least," "average," and "now" (current pain) on a 0–10 numerical rating scale, where 0 indicates no pain and 10 represents the worst pain imaginable. This assessment was conducted weekly during treatment and three months after treatment completion. These single-item measures are supported by IMMPACT recommendations for pain assessment in clinical trials [31,39,40] and the FDA Draft Guidance for Patient-Reported Outcome Measures [41].

iii. The *Brazilian Profile of Chronic Pain: Screen (B-PCP)* identifies the individual's multidimensional pain experience across three domains: pain severity (0-32), disability (0-36), and emotional burden (0-25). Higher scores indicate

greater disability or emotional distress, with a total score ranging from 0 to 93 points. Changes from baseline to the end of treatment and at three months follow-up will be analyzed.

iv. *Fibromyalgia Impact Questionnaire (QIF):* Measures the impact of fibromyalgia on quality of life across three domains: function (9 questions), overall impact (2 questions), and symptoms (10 questions). Scores are weighted differently, with a maximum total score of 100 [42].

v. *Patient Global Impression of Improvement (PGI-I):* A 7-point scale assessing patients' perceptions of symptom improvement post-treatment, ranging from "very much worse" (1) to "very much improved" (7) [43]. Impressions were categorized into "improved" or "very much improved" versus "very much worse" to "slightly improved.

vi. *Quantitative Sensory Testing (QST)* is a standardized protocol using a computer Peltier-based device thermode (measuring 30X30mm), which was attached to the skin on the ventral aspect of the mid-forearm. The temperature was set at 32OC and increased at a rate of 1 OC/s until it reached a maximum of 52Oc. Each participant was instructed to push the button when the stimulation became painful. To determine the heat pain threshold, each patient's threshold was measured three times, with a 40-second break between each assessment. The average of the three assessments was used to define the heat pain threshold for each patient [44].

vii. *The* endogenous pain modulatory system (EPMS) function was assessed through a conditioned pain modulation (CPM) test using a computerized thermode, like the one used to determine the heat pain threshold (HPT). Initially, on the non-dominant forearm, three quantitative sensory testing (QST) assessments were conducted with a 40-second interval between stimuli. Each participant was instructed to press a button when their numerical pain score (NPS, 0-10) reached 6/10. The average temperature of the test stimulus was recorded as T0. After five minutes, participants immersed their dominant hand up to the wrist in water at a temperature of 0°C–1°C for 15 seconds. The QST was then reassessed, resulting in a pain score NPS(T1) based on the evoked pain from the thermode using the T0 temperature on the ventral non-dominant forearm. The CPM test score was calculated as the difference between NPS(T1) and NPS(T0). A negative CPM score indicates proper EPMS function, while a score of ≥ 0 indicates impaired function[45–47].

viii. Side effects of tDCS were evaluated using the Systematic Assessment for Treatment Evaluation questionnaire.

ix. Adherence to the protocol was assessed by the record in the tDCS device, which registers impedance, resistance, time of use, and duration of each session. This data was downloaded at the end of the treatment.

Clinical Measurements: Included assessments of depressive symptoms, central sensitization, sleep quality, analgesic use

x. Demographic Data and Medical Comorbidities: Collected via a standardized questionnaire.

xi. Psychological Assessments: Included the Pittsburgh Sleep Quality Index (PSQI) [48] and the Central Sensitization Inventory for the Brazilian Population (CSI-BP) [49] with scores indicating symptom severity. Depressive symptoms were assessed using the Beck Depression Inventory II[50], and the Pain Catastrophizing Scale (PCS) evaluated catastrophic thinking about pain [51].

xii. Analgesic Use: Patients were allowed to take additional medications as needed and were instructed to record their intake in diaries. The total dose taken each week during the treatment period and three-month follow-up were used for analysis.

Schedule of assessment.

	Baseline	Week1	Week2	Week3	Week4	3-month follow-up
Sociodemographic and lifestyle questionnaire	X					
2016 American College of Rheumatology Diagnostic Criteria (ACR-2016)	X					
Central Sensitization Inventory	X					
Pain Catastrophizing Scale	X				X	
Beck Depression Inventory (BDI-II)	X				X	
Pittsburgh Sleep Quality Index (PSQI)	X				X	
Outcomes	X					
Brief Pain Inventory (BPI)	X	X	X	X	X	X
Patient Global Impression of Improvement (PGI-I)					X	
Fibromyalgia Impact Questionnaire (FIQ)	X				X	
Conditioned Pain Modulation (CPM-test)	X				X	
Side effects of tDCS questionnaire					X	

Schedule of activities

Step Identification	Beginning	Term Screening of participants
Screening of participants	October 9, 2021	March 3 , 2022
Double-blind and randomized phase	April 01, 2022	April 10, 2022
Open follow-up phase	May 15, 2023	
Analysis of data from the randomized phase	July 08, 2024	December 15 2024
Open phase data analysis	February 10, 2024	July 10, 2024

We assure that the FIBRO-HB-tDCS study was initiated only after receiving approval from the CEP-CONEP (Ethical Review Board) System of the Hospital de Clínicas de Porto Alegre, the university hospital of the Federal University of Rio Grande do Sul (UFRGS), Porto Alegre, Brazil.

Statistical Analysis

Continuous and categorical variables were compared using Fisher's exact test, the chi-square test, and the t-test for independent samples. The Shapiro-Wilk normality test was applied to determine the normal distribution of continuous variables. A Linear Mixed-Effects Regression Models for Repeated Measure (LMM) assessed primary outcomes (pain severity and disability) with treatment, placebo response (responder vs. non-responder), time, and the treatment-by-time interaction as fixed effects, and included a random intercept for patients to account for time differences. If appropriate, we then performed the Bonferroni's Test adjustment for post hoc multiple comparisons to identify differences between the groups at each time point and used a paired t test to assess the effects on each experimental group. The Generalized Linear Model (GLM) was used to examine the treatment effect on the secondary outcomes including quality of life, HPT, PGI-I and the descending pain inhibitory system (DPIS). The treatment effect on primary and secondary outcomes was adjusted for analgesic use and placebo susceptibility. Analgesic use was included in the model to control a potential confounder affecting pain outcomes. This ensures that the observed effects of a-tDCS and s-tDCS are attributable to the interventions rather than variations in analgesic use, thereby enhancing the validity of the results. All analyses were adjusted for multiple comparisons using Bonferroni's Test. The modified ITT (m-ITT) analysis included those completing ≥50% of sessions, with missing data imputed using regression model coefficients[16]. In the m-ITT, with an adjusted protocol, encompassing all patients who completed at least ten sessions. The choice of ten sessions for ITT was guided by most of the literature supporting clinical symptom improvement in chronic pain [16,19,52,53]. This method is not as strict as classical ITT, but it suggests that A-tDCS could be useful in clinical settings with at least a minimum number of sessions. Specifically, we replaced missing values with imputed data derived from a regression model using observed data. The treatment group served as the predictor, and the outcome variable with missing values was imputed based on regression coefficients, indicating the relationship between the treatment group and the outcome. This imputation method was applied, for example, to ten patients who withdrew after ten tDCS sessions. Sensitivity analyses confirmed consistent LMM results with or without imputation (*see per-protocol analysis in results - Supplement II*).

The standardized difference means (SDM) was used to compute the effect size (ES) by dividing the mean difference between a-tDCS and s-tDCS by the pooled baseline standard deviation (SD). The ES was interpreted as small if lower than 0.20 to 0.49, moderate if between 0.50 and 0.79, and large if larger than 0.80 [54]. All statistical analyses were performed using two-tailed tests at the 5% significance level with SPSS, version 22.0 (SPSS, Chicago, IL).

Risks and Benefits of the Study:

The study involved minimal risk. The two procedures—multisession (a-tDCa) targeting the PFC, combined with exercise (E) and pain education (PEN)—had been previously studied in home settings, with no reports of serious complications or brain damage. tDCS used a low-intensity current, which was safe for fibromyalgia, with the most common side effect being mild scalp tingling, minimized by humidifying the electrodes with saline.

The main benefit for participants was the potential improvement in pain-related symptoms of fibromyalgia. Participants could also join a 3-month follow-up phase, where a-tDCS -E-PEN was offered in an open phase. Although the study took

place at home, patients visited the research center for periodic clinical evaluations and had remote access to the research team for questions and updates. Any clinical complications were treated at the Hospital de Pronto Socorro, Porto Alegre, Brazil, with treatment provided free of charge as needed.

After the study, participants were referred back to their original outpatient clinics with appropriate prescriptions and care instructions

Study Discontinuation Criteria

The researchers had the prerogative to cease study participation if any clinical condition emerged that could endanger the patient's health or life. In the case of FIBRO-HB-tDCS, special attention was given to the emergence of suicidal ideation and/or planning, and (hypo)manic episodes, as depression is found in approximately 60% of fibromyalgia cases. In such situations, the participant was excluded from the study, regardless of when the issue arose, and referred for appropriate treatment.

Study Results

We ensured that the results of the study were disseminated to the broader community through appropriate channels for both the scientific community and the participating patients. It was clearly explained that the research was conducted at the Hospital de Clínicas de Porto Alegre, Porto Alegre, Brazil.

p. Dissemination of Results

We guarantee that the results of our research were submitted for publication, with full credit given to all authors involved.

o. REFERENCES

1. Jones GT, Atzeni F, Beasley M, Flüß E, Sarzi-Puttini P, Macfarlane GJ. The prevalence of fibromyalgia in the general population: a comparison of the American College of Rheumatology 1990, 2010, and modified 2010 classification criteria. *Arthritis Rheumatol Hoboken NJ*. 2015;67(2):568-575. doi:10.1002/art.38905
2. Desai R, Jo A, Marlow NM. Risk for Medication Nonadherence Among Medicaid Enrollees With Fibromyalgia: Development of a Validated Risk Prediction Tool. *Pain Pract Off J World Inst Pain*. 2019;19(3):295-302. doi:10.1111/papr.12743
3. Cui Z, Zhao Y, Novick D, Faries D. Predictors of duloxetine adherence and persistence in patients with fibromyalgia. *J Pain Res*. 2012;5:193-201. doi:10.2147/JPR.S31800
4. Moore RA, Fisher E, Häuser W, et al. Pharmacological therapies for fibromyalgia (fibromyalgia syndrome) in adults - an overview of Cochrane Reviews - Moore, RA - 2021 | Cochrane Library. Accessed September 30, 2024. https://www.cochranelibrary.com/cdsr/doi/10.1002/14651858.CD013151.pub2/full
5. Macfarlane GJ, Kronisch C, Dean LE, et al. EULAR revised recommendations for the management of fibromyalgia. *Ann Rheum Dis*. 2017;76(2):318-328. doi:10.1136/annrheumdis-2016-209724
6. Petzke F, Brückle W, Eidmann U, et al. [General treatment principles, coordination of care and patient education in fibromyalgia syndrome : Updated guidelines 2017 and overview of systematic review articles]. *Schmerz Berl Ger*. 2017;31(3):246-254. doi:10.1007/s00482-017-0201-6
7. Perrot S, Russell IJ. More ubiquitous effects from non-pharmacologic than from pharmacologic treatments for fibromyalgia syndrome: a meta-analysis examining six core symptoms. *Eur J Pain Lond Engl*. 2014;18(8):1067-1080. doi:10.1002/ejp.564
8. Carvalho F, Brietzke AP, Gasparin A, et al. Home-Based Transcranial Direct Current Stimulation Device Development: An Updated Protocol Used at Home in Healthy Subjects and Fibromyalgia Patients. *J Vis Exp JoVE*. 2018;(137):57614. doi:10.3791/57614
9. Lefaucheur JP, Wendling F. Mechanisms of action of tDCS: A brief and practical overview. *Neurophysiol Clin Clin Neurophysiol*. 2019;49(4):269-275. doi:10.1016/j.neucli.2019.07.013

10. Huang Y, Thomas C, Datta A, Parra LC. Optimized tDCS for Targeting Multiple Brain Regions: An Integrated Implementation. *Annu Int Conf IEEE Eng Med Biol Soc IEEE Eng Med Biol Soc Annu Int Conf*. 2018;2018:3545-3548. doi:10.1109/EMBC.2018.8513034
11. Fregni F, El-Hagrassy MM, Pacheco-Barrios K, et al. Evidence-Based Guidelines and Secondary Meta-Analysis for the Use of Transcranial Direct Current Stimulation in Neurological and Psychiatric Disorders. *Int J Neuropsychopharmacol*. 2021;24(4):256-313. doi:10.1093/ijnp/pyaa051
12. Polanía R, Nitsche MA, Korman C, Batsikadze G, Paulus W. The importance of timing in segregated theta phase-coupling for cognitive performance. *Curr Biol CB*. 2012;22(14):1314-1318. doi:10.1016/j.cub.2012.05.021
13. Caumo W, Alves RL, Vicuña P, et al. Impact of Bifrontal Home-Based Transcranial Direct Current Stimulation in Pain Catastrophizing and Disability due to Pain in Fibromyalgia: A Randomized, Double-Blind Sham-Controlled Study. *J Pain*. 2022;23(4):641-656. doi:10.1016/j.jpain.2021.11.002
14. Charvet LE, Shaw MT, Bikson M, Woods AJ, Knotkova H. Supervised transcranial direct current stimulation (tDCS) at home: A guide for clinical research and practice. *Brain Stimulat*. 2020;13(3):686-693. doi:10.1016/j.brs.2020.02.011
15. Brietzke AP, Zortea M, Carvalho F, et al. Large Treatment Effect With Extended Home-Based Transcranial Direct Current Stimulation Over Dorsolateral Prefrontal Cortex in Fibromyalgia: A Proof of Concept Sham-Randomized Clinical Study. *J Pain*. 2020;21(1-2):212-224. doi:10.1016/j.jpain.2019.06.013
16. Caumo W, Ramos RL, Serrano PV, et al. Efficacy of Home-Based Transcranial Direct Current Stimulation Over the Primary Motor Cortex and Dorsolateral Prefrontal Cortex in the Disability Due to Pain in Fibromyalgia: A Factorial Sham-Randomized Clinical Study. *J Pain*. 2024;25(2):376-392. doi:10.1016/j.jpain.2023.09.001
17. Valero-Cabré A, Amengual JL, Stengel C, Pascual-Leone A, Coubard OA. Transcranial magnetic stimulation in basic and clinical neuroscience: A comprehensive review of fundamental principles and novel insights. *Neurosci Biobehav Rev*. 2017;83:381-404. doi:10.1016/j.neubiorev.2017.10.006
18. Moriarty TA, Mermier C, Kravitz L, Gibson A, Beltz N, Zuhl M. Acute Aerobic Exercise Based Cognitive and Motor Priming: Practical Applications and Mechanisms. *Front Psychol*. 2019;10. doi:10.3389/fpsyg.2019.02790
19. Cardenas-Rojas A, Pacheco-Barrios K, Giannoni-Luza S, Rivera-Torrejon O, Fregni F. Noninvasive brain stimulation combined with exercise in chronic pain: a systematic review and meta-analysis. *Expert Rev Neurother*. 2020;20(4):401-412. doi:10.1080/14737175.2020.1738927
20. Nitsche MA, Paulus W. Excitability changes induced in the human motor cortex by weak transcranial direct current stimulation. *J Physiol*. 2000;527(Pt 3):633-639. doi:10.1111/j.1469-7793.2000.t01-1-00633.x
21. Im JJ, Jeong H, Bikson M, et al. Effects of 6-month at-home transcranial direct current stimulation on cognition and cerebral glucose metabolism in Alzheimer's disease. *Brain Stimulat*. 2019;12(5):1222-1228. doi:10.1016/j.brs.2019.06.003
22. Boggio PS, Nunes A, Rigonatti SP, Nitsche MA, Pascual-Leone A, Fregni F. Repeated sessions of noninvasive brain DC stimulation is associated with motor function improvement in stroke patients. *Restor Neurol Neurosci*. 2007;25(2):123-129.
23. Song S, Zilverstand A, Gui W, Li HJ, Zhou X. Effects of single-session versus multi-session non-invasive brain stimulation on craving and consumption in individuals with drug addiction, eating disorders or obesity: A meta-analysis. *Brain Stimulat*. 2019;12(3):606-618. doi:10.1016/j.brs.2018.12.975
24. Sandran N, Hillier S, Hordacre B. Strategies to implement and monitor in-home transcranial electrical stimulation in neurological and psychiatric patient populations: a systematic review. *J NeuroEngineering Rehabil*. 2019;16(1):58. doi:10.1186/s12984-019-0529-5
25. Antal A, Terney D, Kühnl S, Paulus W. Anodal Transcranial Direct Current Stimulation of the Motor Cortex Ameliorates Chronic Pain and Reduces Short Intracortical Inhibition. *J Pain Symptom Manage*. 2010;39(5):890-903. doi:10.1016/j.jpainsymman.2009.09.023
26. Pilloni G, Shaw M, Feinberg C, et al. Long term at-home treatment with transcranial direct current stimulation (tDCS) improves symptoms of cerebellar ataxia: a case report. *J Neuroengineering Rehabil*. 2019;16(1):41. doi:10.1186/s12984-019-0514-z
27. Serrano PV, Zortea M, Alves RL, et al. The effect of home-based transcranial direct current stimulation in cognitive performance in fibromyalgia: A randomized, double-blind sham-controlled trial. *Front Hum Neurosci*. 2022;16. doi:10.3389/fnhum.2022.992742
28. Brietzke AP, Antunes LC, Carvalho F, et al. Potency of descending pain modulatory system is linked with peripheral sensory dysfunction in fibromyalgia: An exploratory study. *Medicine (Baltimore)*. 2019;98(3):e13477. doi:10.1097/MD.0000000000013477
29. Keller S, Bann CM, Dodd SL, Schein J, Mendoza TR, Cleeland CS. Validity of the brief pain inventory for use in documenting the outcomes of patients with noncancer pain. *Clin J Pain*. 2004;20(5):309-318. doi:10.1097/00002508-200409000-00005
30. Krebs EE, Bair MJ, Damush TM, Tu W, Wu J, Kroenke K. Comparative responsiveness of pain outcome measures among primary care patients with musculoskeletal pain. *Med Care*. 2010;48(11):1007-1014. doi:10.1097/MLR.0b013e3181eaf835
31. Dworkin RH, Turk DC, Wyrwich KW, et al. Interpreting the clinical importance of treatment outcomes in chronic pain clinical trials: IMMPACT recommendations. *J Pain*. 2008;9(2):105-121. doi:10.1016/j.jpain.2007.09.005
32. Borges RB, Mancuso ACB, Camey SA, et al. Power and Sample Size for Health Researchers: uma ferramenta para cálculo de tamanho amostral e poder do teste voltado a pesquisadores da área da saúde. *Clin Biomed Res*. 2020;40(4). Accessed May 4, 2023. https://seer.ufrgs.br/index.php/hcpa/article/view/109542
33. Bikson M, Grossman P, Thomas C, et al. Safety of Transcranial Direct Current Stimulation: Evidence Based Update 2016. *Brain*

Stimulat. 2016;9(5):641-661. doi:10.1016/j.brs.2016.06.004
34. Rondon-Ramos A, Martinez-Calderon J, Diaz-Cerrillo JL, et al. Pain Neuroscience Education Plus Usual Care Is More Effective Than Usual Care Alone to Improve Self-Efficacy Beliefs in People with Chronic Musculoskeletal Pain: A Non-Randomized Controlled Trial. *J Clin Med*. 2020;9(7):2195. doi:10.3390/jcm9072195
35. Watson JA, Ryan CG, Cooper L, et al. Pain Neuroscience Education for Adults With Chronic Musculoskeletal Pain: A Mixed-Methods Systematic Review and Meta-Analysis. *J Pain*. 2019;20(10):1140.e1-1140.e22. doi:10.1016/j.jpain.2019.02.011
36. Caneiro JP, Bunzli S, O'Sullivan P. Beliefs about the body and pain: the critical role in musculoskeletal pain management. *Braz J Phys Ther*. 2021;25(1):17-29. doi:10.1016/j.bjpt.2020.06.003
37. Leventhal H, Phillips LA, Burns E. The Common-Sense Model of Self-Regulation (CSM): a dynamic framework for understanding illness self-management. *J Behav Med*. 2016;39(6):935-946. doi:10.1007/s10865-016-9782-2
38. Brief Pain Inventory (BPI). MD Anderson Cancer Center. Accessed September 30, 2024. https://www.mdanderson.org/research/departments-labs-institutes/departments-divisions/symptom-research/symptom-assessment-tools/brief-pain-inventory.html
39. Dworkin RH, Turk DC, Farrar JT, et al. Core outcome measures for chronic pain clinical trials: IMMPACT recommendations. *Pain*. 2005;113(1-2):9-19. doi:10.1016/j.pain.2004.09.012
40. Turk DC, Dworkin RH, Burke LB, et al. Developing patient-reported outcome measures for pain clinical trials: IMMPACT recommendations. *Pain*. 2006;125(3):208-215. doi:10.1016/j.pain.2006.09.028
41. FDA C for DE and. The FDA Announces New Prescription Drug Information Format. *FDA*. Published online 2006. Accessed September 30, 2024. https://www.fda.gov/drugs/laws-acts-and-rules/fda-announces-new-prescription-drug-information-format
42. Marques AP, Santos AMB, Assumpção A, Matsutani LA, Lage LV, Pereira CAB. Validação da versão brasileira do Fibromyalgia Impact Questionnaire (FIQ). *Rev Bras Reumatol*. 2006;46:24-31. doi:10.1590/S0482-50042006000100006
43. Geisser ME, Palmer RH, Gendreau RM, Wang Y, Clauw DJ. A pooled analysis of two randomized, double-blind, placebo-controlled trials of milnacipran monotherapy in the treatment of fibromyalgia. *Pain Pract Off J World Inst Pain*. 2011;11(2):120-131. doi:10.1111/j.1533-2500.2010.00403.x
44. Schestatsky P, Stefani LC, Sanches PR, et al. Validation of a Brazilian quantitative sensory testing (QST) device for the diagnosis of small fiber neuropathies. *Arq Neuropsiquiatr*. 2011;69(6):943-948. doi:10.1590/s0004-282x2011000700019
45. de Oliveira Franco Á, da Silveira Alves CF, Vicuña P, et al. Hyper-connectivity between the left motor cortex and prefrontal cortex is associated with the severity of dysfunction of the descending pain modulatory system in fibromyalgia. *PloS One*. 2022;17(5):e0247629. doi:10.1371/journal.pone.0247629
46. Soldatelli MD, Siepmann T, Illigens BMW, et al. Mapping of predictors of the disengagement of the descending inhibitory pain modulation system in fibromyalgia: an exploratory study. *Br J Pain*. 2021;15(2):221-233. doi:10.1177/2049463720920760
47. Granovsky Y. Conditioned pain modulation: a predictor for development and treatment of neuropathic pain. *Curr Pain Headache Rep*. 2013;17(9):361. doi:10.1007/s11916-013-0361-8
48. Bertolazi AN, Fagondes SC, Hoff LS, et al. Validation of the Brazilian Portuguese version of the Pittsburgh Sleep Quality Index. *Sleep Med*. 2011;12(1):70-75. doi:10.1016/j.sleep.2010.04.020
49. Caumo W, Antunes LC, Elkfury JL, et al. The Central Sensitization Inventory validated and adapted for a Brazilian population: psychometric properties and its relationship with brain-derived neurotrophic factor. J Pain Res. 2017;10:2109-2122. doi:10.2147/JPR.S131479
50. Gorenstein C, Andrade L. Validation of a Portuguese version of the Beck Depression Inventory and the State-Trait Anxiety Inventory in Brazilian subjects. *Braz J Med Biol Res Rev Bras Pesqui Medicas E Biol*. 1996;29(4):453-457.
51. Sehn F, Chachamovich E, Vidor LP, et al. Cross-cultural adaptation and validation of the Brazilian Portuguese version of the pain catastrophizing scale. *Pain Med Malden Mass*. 2012;13(11):1425-1435. doi:10.1111/j.1526-4637.2012.01492.x
52. Jornada MN da, Antunes LC, Alves C, et al. Impact of multiple-session home-based transcranial direct current stimulation (M-HB-tDCS) on eating behavior in fibromyalgia: A factorial randomized clinical trial. *Brain Stimulat*. 2024;17(2):152-162. doi:10.1016/j.brs.2024.02.001
53. Teixeira PEP, Tavares DRB, Pacheco-Barrios K, et al. Development of a Clinical Prediction Rule for Treatment Success with Transcranial Direct Current Stimulation for Knee Osteoarthritis Pain: A Secondary Analysis of a Double-Blind Randomized Controlled Trial. *Biomedicines*. 2022;11(1):4. doi:10.3390/biomedicines11010004
54. Kazis LE, Anderson JJ, Meenan RF. Effect sizes for interpreting changes in health status. *Med Care*. 1989;27(3 Suppl):S178-189. doi:10.1097/00005650-198903001-00015
