# Supplementary material for: Home-Based Transcranial Direct Current Stimulation vs Placebo for Fibromyalgia: A Randomized Clinical Trial
Source: JAMA Netw Open. 2025 Jun 6;8(6):e2514262. doi: 10.1001/jamanetworkopen.2025.14262 (PMC12144624; doi:10.1001/jamanetworkopen.2025.14262)
Supplement: Supplement 2. — eMethods. eTable 1. Multidimensional Pain Interference Index: Per Protocol Analysis of Interventions eTable 2. Pain Severity (Average and Worst Pain) and Disability Due to Pain (B-PCP Total Score) eTable 3. LMM Analysis of the Intervention Groups (a-tDCS vs s-tDCS) on BPI Pain Severity and B-PCP Total Score, Including Their Interactions With Time and Placebo-Test Response (n = 112) eTable 4. Patient Impression of Improvement eTable 5. Side Effects Presented as Percentage, and the Incidence or Severity of Side Effects Classified as Absence, Mild, Moderate, and Severe (n = 112) [file jamanetwopen-e2514262-s002.pdf]

Supplemental Online Content

Caumo W, Franca BR, Orzechowski R, et al. Home-based transcranial direct current stimulation vs placebo for fibromyalgia: a randomized clinical trial. *JAMA Netw Open*. 2025;8(6):e2514262. doi:10.1001/jamanetworkopen.2025.14262

eMethods.

eTable 1. Multidimensional Pain Interference Index: Per Protocol Analysis of Interventions

eTable 2. Pain Severity (Average and Worst Pain) and Disability Due to Pain (B-PCP Total Score)

eTable 3. LMM Analysis of the Intervention Groups (a-tDCS vs. s-tDCS) on BPI Pain Severity and B-PCP Total Score, Including Their Interactions With Time and Placebo-Test Response (n=112)

eTable 4. Patient Impression of Improvement

eTable 5. Side Effects Presented as Percentage, and the Incidence or Severity of Side Effects Classified as Absence, Mild, Moderate, and Severe (n=112)

This supplemental material has been provided by the authors to give readers additional information about their work.

© 2025 Caumo W et al. *JAMA Network Open*

eMethods.

Primary outcome – Per Protocol Analysis

Effects of treatment assessed by the Multidimensional Pain Interference Index

Linear Mixed Model (LMM) analysis showed a significant MPII reduction for a-tDCS vs. s-tDCS across treatment and follow-up (F=10.52, P<0.01), with a time effect F=11.36, P<0.01, but not no Time vs. group (F=1.75, p=0.04) e interaction (Table 1). A placebo effect was observed (F = 5.14, p < 0.02) with no interaction with treatment **(**β = -0.21, 95% CI: -0.93 to 0.51, p = 0.56**).** This significant reduction in pain interference highlights the efficacy of the active intervention, providing considerable advantages over the sham group. Higher pain interference in daily activities correlated with greater analgesic use (β=0.29, 95% CI: 0.14 to 0.44, p<0.01).

The estimated marginal means (EMM) were 4.06 (SD=1.11) for the a-tDCS group and 5.00 (SD=1.20) for the control group, indicating a large effect size (ES=0.82, mean difference=0.94, pooled SD=1.15). From baseline to the end of treatment, the a-tDCS group showed a reduction of 35.81% (29.24 to 42.40), compared to 15.87% (9.38 to 22.36) in the s-tDCS group. This significant reduction in pain interference highlights the efficacy of the active intervention, providing considerable advantages over the sham group.

The analysis based on placebo-test responses (responders vs. non-responders) showed a larger ES of a-tDCS in responders. In responders, EMM for MPII was 4.09 (1.11) for a-tDCS vs. 5.06 (1.14) for s-tDCS (F = 4.35, p<0.01), with a mean difference of 0.97 (pooled SD: 1.12) and a large ES (0.88). In non-responders, EMM was 3.93 (1.11) for a-tDCS vs. 4.63 (1.10) for s-tDCS (F = 9.16, p<0 01), with a mean difference of 0.7 (pooled SD: 1.10) and a moderate ES (0.64).

© 2025 Caumo W et al. *JAMA Network Open*

e-1.1. eTable 1. Multidimensional Pain Interference Index: Per protocol analysis of Interventions.

Data Presented as Mean (Standard Deviation, SD) and relative change from baseline (%), with 95% CI, confidence intervals (n = 102).

	**Per protocol (n=102)®**			
**Primary Outcome**	**s-tDCS**		**a-tDCS**	
**Change in BPI-pain interference**	**(n=50)**		**(n=52)**	
	Mean (SD)	Change (%), CI 95%)	Mean (SD)	Change (%), CI 95%)
Baseline	5.96 (2.03) [&]		5.79 (1.61) [&]	
Week 1	4.63(2.17)	-15.49 (-27.63 to -3.03)	4.31 (1.87)	-27.83 (-42.95 to -18.64)
Week 2	4.69 (1.03)	-21.04 (-30.89 to -11.15)	3.69 (1.88)	-36.94 (-45.94 to -24.47)
Week 3	5.33 (1.21)	-11.05 (-33.95 to -11.85)	3.68 (1.67)	-36.02 (-58.70 to -9.98)
Week 4 (End of treatment)	4.81 (1.19)	-16.92 (-28.75 to -5.08)	3.00 (2.23)	-50.19 (-62.08 to -39.86)
3-month follow-up	4.58 (1.36)	-14.94 (-28.02 to -1.85)	3.82 (1.38)	-29.46 (-42.32 to -16.53)
		-15.87 (-22.36 to -9.38)		-35.81 (-42.40 to -29.24)
Group (F=12.69, P<0.001);		*Time (F=13.75, P<0.001);*	*Time vs. group (F=2.31, p=0.04)*	

[&] *Reference of category*
Relative change from baseline is expressed as a percentage (%) change from baseline. 95% Confidence Interval (CI 95%)

e.2. Secondary outcomes

BPI Pain Severity Assessed by Average and the Worst Pain in the Last 24 Hours and disability due to pain assessed by the B-PCS total score

The e.2.1. Table 2 displays the relative change in scores from baseline to each assessment point. The data are presented in table **e.2.1. Table 2.**

© 2025 Caumo W et al. *JAMA Network Open*

e.2.1. eTable 2. Pain severity (average and worst pain) and disability due to pain (B-PCP Total Score). Data are presented as mean, standard deviation (SD), and relative change from baseline (%), with 95% CI, confidence intervals to each assessment point throughout the protocol (n = 112).

**Primary Outcome**	**s-tDCS (n=56)**		**a-tDCS (n=56)**	
**BPI-pain severity**		**Change (%) CI 95%**		**Change (%) CI 95%**
Change in BPI- pain average in the last 24 hours				
Baseline	5.96 (1.93)[&]		6.30 (1.54) [&]	
Week 1	5.05 (1.95)	-33.49 (-53.55 to -13.43)	4.79 (2.13)	-56.86 (-76.37 to -37.35)
Week 2	4.82(1.85)	-36.32 (-56.38 to -16.26)	4.75 (1.76)	-55.03 (-74.54 -35.53)
Week 3	4.75 (2.13)	-36.54 (-55.87 to -17.20)	3.70 (1.73)	-75.06 (-95.12 to -55.00)
Week 3 (End of treatment)	4.11 (1.73)	-52.39 (-72.08 to -32.70)	3.81 (1.48)	-83.12 (-91.80 to -73.43)
3-month follow-up	5.11 (2.46)	-24.22 (-43.91 to -4.54)	4.65 (1.68)	-53.09 (-72.78 to -33.41)
Change in BPI- worst pain in the last 24 hours				
Baseline	6.89 (2.09) [&]		7.32 (1.59) [&]	
Week 1	5.87 (1.99)	-33 (-54.86 to -11.68)	5.82 (1.91)	-43 (-64.45 to -22.07)
Week 2	6.14 (2.38)	-21.98 (-44.21 to 5.75)	5.71 (1.66	-54.95 (-76.14 to -33.75)
Week 3	5.59 (2.48)	-42.45 (-64.24 to -20.69)	4.88 (2.37)	-70.96 (-93.19 to -48.73)
Week 3 (End of treatment)	5.62 (2.29)	-35.17 (-56.18 to -14.17)	4.82 (2.14)	-48.36 (-69.56 to -27.17)
3-month follow-up	5.71 (3.0)	-40.73 (-61.92 to -19.54)	4.67(1.86)	-65.34 (-86.73 to -43.95)
Profile of chronic pain: screen for a Brazilian population (B-PCP:S) –total score				
Baseline	72.66 (13.29) [&]		72.68 (11.11) [&]	
End of treatment	62.66 (16.38)	-20.83 (-31.74 to - 9.93)	58.75 (19.23)	-40.85 (-51.75 to -29.94)
3-month follow-up	63.45 (14.90)	-18.83 (-29.74 to -7.93)	56.49 (17.77)	-39.43 (-50.55 to -28.32)

The LMM analysis of secondary outcomes (Table 3) assessed the effects of s-tDCS and a-tDCS over time, accounting for time effects, time × intervention interaction, analgesic use, and placebo-test response. Higher pain scores (BPI pain severity, B-PCP:S total) correlated with increased analgesic use. While the interaction analysis suggested a potential placebo effect, it was not statistically significant.

The LMM results showed that a-tDCS significantly reduced BPI pain severity and disability (B-PCP total) compared to s-tDCS. A significant time effect was observed, but no time × intervention interaction was found for any outcome.

© 2025 Caumo W et al. *JAMA Network Open*

e.2.2. eTable 3. LMM analysis of the intervention groups (a-tDCS vs. s-tDCS) on BPI pain severity and B-PCP total score, including their interactions with time and placebo-test response (n=112).

BPI- Pain severity				
BPI- pain average in the last 24 hours	**Estimate**	**SEM**	**P-value**	**CI (95%)**
Intercept	3.803	.318	<.00	(3.17 to 4.43)
a-tDCS / s-tDCS	1.254	.460	<.00	(.34 to 2.16)
Placebo-test responders (No/Yes)	-.179	.216	.40	(-.60 to .24)
BPI- Pain interference at treatment end vs. T0 as reference	-2,830	.325	<.00	(-3.47 to -2.10)
BPI- Pain interference at 3rd month of follow-up vs. T0 as reference	-2.310	.375	<.00	(-3.05 to -1.57)
Analgesic use across treatment and follow-up	.233	.051	<.00	(.13 to 0.33)
Interaction analysis				
a-tDCS / s-tDCS *vs.* Placebo-test responders (No/Yes)	-.566	.307	.06	(-1.16 to 0.04)
a-tDCS / s-tDCS- at treatment end *vs.* T0 as reference	. 865	.465	.04	(.05 to 1.78)
a-tDCS / s-tDCS -at 3rd month of follow-up *vs.* T0 as reference	1.222	.536	.02	(.16 to 2.27)
Effect of Groups (F=4.94 , P=0.02); Time (F=21.12, P<0.02); Time vs. group (F=0.39, P=085)				
Pain severity – Worst pain in the last 24 hours				
Intercept	5.821	.339	.00	(5.15 to 6.49)
a-tDCS / s-tDCS	.605	.492	.22	(-.36 to1.57)
Placebo-test responders (No/Yes)	-.166	.246	.49	(-.65 to 0.31)
BPI- Pain interference at treatment end vs. T0 as reference	-2.482	.403	<.00	(-3.27 to -1.68)
BPI- Pain interference at 3rd month of follow-up vs. T0 as reference	-1.338	.400	<.00	(-2.12 to -0.54)
Analgesic use across treatment and follow-up	-.069	.079	.37	(-.22 to 0.08)
Interaction analysis				
a-tDCS / s-tDCS *vs.* Placebo-test responders (No/Yes)	-.274	.349	.43	(-.96 to 0.41)
a-tDCS / s-tDCS- at treatment end *vs.* T0 as reference	1.569	.577	<.00	(.43 to 2.70)
a-tDCS / s-tDCS -at 3rd month of follow-up *vs.* T0 as reference	.813	.571	.15	(-.31 to 1.93)
Effect of Groups (F=5.14 , P=0.02); Time (F=25.8, P<0.01); Time vs. group (F=1.74, P=0.12))				
Profile of chronic pain: screen for a Brazilian population (B-PCP:S) –total score				
Intercept	52.788	2.912	<.00	(47.03 to 58.54)
a-tDCS / s-tDCS	12.774	4.130	<.00	(4.60 to 20.94)
Placebo-test responders (No/Yes)	3.043	2.517	.22	(-1.91 to 7.99)
B-PCP:S at treatment end vs. T0 as reference	-9.951	2.163	<.00	(-1517 to -4.72)
B-PCP:S at 3rd month of follow-up vs. T0 as reference	-12.883	2.190	<.00	(-18.17 to -7.59)
Analgesic use across treatment and follow-up	1.672	.5257	<.00	(.63 to 2.70)
Interaction analysis				
a-tDCS / s-tDCS *vs.* Placebo-test responders (No/Yes)	6.824	3.523	.054	(-.11 to 13.75)
a-tDCS / s-tDCS- at treatment end *vs.* T0 as reference	3.872	4.974	.437	(-5.93 to 13.67)
a-tDCS / s-tDCS -at 3rd month of follow-up *vs.* T0 as reference	7.839	4.370	.075	(-.78 to 16.46)
Effect of Groups (F=5.96 , P=0.01); Time (F=20.590, P<0.01); Time vs. group (F=1.36, P=0.26)				

B-PCP:S - Brazilian Portuguese version of the Profile of Chronic Pain: Screen total score.

Degrees of freedom (Df). Anodal-(a) transcranial direct current stimulation (a-tDCS) or sham stimulation (s-tDCS) combined with exercise (E) and pain education guidance (PEG). Assessment at baseline (time zero, T0)

© 2025 Caumo W et al. *JAMA Network Open*

e.2.3. Improvement in Patient Impressions and Reduction in Pain Interference

Patients treated with a-tDCS showed significant improvement, with 84.9% reporting they were much or moderately improved, compared to 55% in the s-tDCS group. The sham group had a relative risk of 2.048 (95% CI, 1.42–2.95) of reporting only slight improvement or no change, representing a 104.8% increased risk compared to the active treatment group. In ***e.2.3. Table 4***, the patients' impressions of improvement are presented based on the categories of the PGI-I scale.

e.2.3. eTable 4. Patient Impression of Improvement. Data are presented as absolute numbers and percentage (n=112)

Response Category	s-tDCS (n=56)		a-tDCS (n=56)		Pearson χ^2	Df	P-value
Much Improved	**3**	**(5.4%)**	**13**	**(23.2%)**	**15.66**	**1**	**<.00**
Moderately Improved	**22**	**(39.3%)**	**32**	**(57.1%)**			
Slightly Improved	**25**	**(44.6%)**	**5**	**(8.9%)**			
No Change	**3**	**(5.4%)**	**4**	**(7.1%)**			

e.2.4. Assessment of Adverse Events and Safety

The most frequent side effects in both groups were pain in the stimulation area (71.5% in both), tingling (50.4% a-tDCS vs. 54.2% s-tDCS), and burning (62.1% a-tDCS vs. 48.1% s-tDCS). Headache was slightly more common in the s-tDCS group (40.6% vs. 29.9%), while sleepiness (41.1% a-tDCS vs. 32.2% s-tDCS) and mood changes (59.8% a-tDCS vs. 49.5% s-tDCS) were reported more frequently in the a-tDCS group. Most symptoms were mild to moderate in both groups.

© 2025 Caumo W et al. *JAMA Network Open*

e.2. eTable 5. Side effects presented as percentage, and the incidence or severity of side effects classified as absence, mild, moderate, and severe (n=112)

Symptoms	Group	Absence	Mild	Moderate	Severe	P-value
Headache	(1) a-tDCS (n=56)	64 (29.9%)	70 (32.7%)	63 (29.4%)	13 (15.3%)	0.10
	(2) s-tDCS (n=56)	91 (40.6%)	54 (24.1%)	63 (29.5%)	17 (14.7%)	
Neck pain	(1) a-tDCS (n=56)	94 (42%)	40 (17.9%)	61 (27.2%)	29 (12.9%)	0.23
	(2) s-tDCS (n=56)	61(28.5%)	63(29.4%)	70 (32.7%)	20 (9.3%)	
Pain the area of stimulation	(1) a-tDCS (n=56)	153 (71.5%)	45 (20.1%)	24 (10.7%)	2 (.9%)	0.97
	(2) s-tDCS (n=56)	153 (71.5%)	37 (17.3%)	15 (7%)	9 (4.2%)	
Tingling	(1) a-tDCS (n=56)	113 (50.4%)	71 (67.5%)	36 (16.1%)	4 (6.1%)	0.78
	(2) s-tDCS (n=56)	116 (54.2%)	61 (28.5%)	29 (31.8%)	8 (5.9%)	
Itching	(1) a-tDCS (n=56)	102 (45.5%)	69 (30.8%)	48 (21.4%)	5 (6.6%)	0.56
	(2) s-tDCS (n=56)	89 (41.9%)	76 (35.5%)	41 (19.2%)	8 (6.4%)	
Burning	(1) a-tDCS (n=56)	139 (62.1%)	56 (25%)	27 (12.1%)	2 (0.9%)	0.004
	(2) s-tDCS (n=56)	103 (48.1%)	72 (33.6%)	32 (15%)	7 (4.4%)	
Redness	(1) a-tDCS (n=56)	203 (90.6%)	14 (11.3%)	6 (9.2%)	1 (0.4%)	0.139
	(2) s-tDCS (n=56)	190 (88.8%)	8 (10.7%)	12 (8.8%)	4 (1.9%)	
Sleepiness	(1) a-tDCS (n=56)	92 (41.1%)	53 (23.7%)	64 (28.6%)	15 (6.7%)	0.08
	(2) s-tDCS (n=56)	69 (32.25%)	59 (27.6%	66 (30.8%)	20 (9.3%)	
Trouble of concentration	(1) a-tDCS (n=56)	85 (37.9%)	49 (21.9%)	79 (35.3%)	11 (%)9.8	0.24
	(2) s-tDCS (n=56)	61 (28.5%)	75 (35%)	57 (28.6%)	21 (4.9%)	
Humor changes	(1) a-tDCS (n=56)	134 (59.8%)	40 (17.9%)	34 (15.2%)	16 (7.1%)	0.06
	(2) s-tDCS (n=56)	106 (49.5%)	48 (22.4%	41 (19.4%)	19 (8.9%)	
