# Supplementary material for: Home-Based Transcranial Direct Current Stimulation vs Placebo for Fibromyalgia: A Randomized Clinical Trial
Source: JAMA Netw Open. 2025 Jun 6;8(6):e2514262. doi: 10.1001/jamanetworkopen.2025.14262 (PMC12144624; doi:10.1001/jamanetworkopen.2025.14262)
Supplement: Supplement 3. — Video Daily Exercise Program Used Before Transcranial Direct Current [file jamanetwopen-e2514262-s003.pdf]

Supplemental Online Content

Caumo W, Franca BR, Orzechowski R, et al. Home-based transcranial direct current stimulation vs placebo for fibromyalgia: a randomized clinical trial. *JAMA Netw Open*. 2025;8(6):e2514262. doi:10.1001/jamanetworkopen.2025.14262

Video Daily Exercise Program Used Before Transcranial Direct Current

This supplemental material has been provided by the authors to give readers additional information about their work.

© 2025 Caumo W et al. *JAMA Network Open*

Video 4 – Daily Exercise Program Used Before Transcranial Direct Current Stimulation

English Translation with time stamps for the speaking part

0:00 In this video I will show you
0:02 a sequence of exercises that
0:04 you must do from Monday to
0:06 Friday before using the electrostimulation cap.
0:09 You will do two sets
0:12 of ten repetitions. In each set
0:16 one-minute break between each series, ok?
0:19 Remember to find a comfortable position
0:22 sitting or standing and
0:27 if necessary, use a back support
0:30 A pillow a cushion and the feet should not
0:33 be left rigid right, loose in the air;
0:36 they must have contact to the floor
0:39 during the execution of the exercises
0:42 sitting. In this movement you will
0:45 bend your arm towards your shoulder
0:49 Ok? Make a slow movement, don't
0:52 forget to breathe and look for a
0:56 comfortable place, in case you can't
0:59 put your feet on the floor while sitting
1:02 get support for your feet
1:08 In this movement you will
1:11 extend a leg at a time and you
1:14 will make five times for each leg
1:17 Rest a minute and then five more times
1:20 for each leg.
1:24 This is the opening movement of the
1:27 arms right? Look for a place with space
1:31 Make ten movements. Rest one a minute and
1:34 Do ten more movements
1:38 Always slowly and breathing
1:42 This is the movement where you will
1:44 strengthen the musculature of your leg
1:46 It is a hip flexion movement. Lean on a
1:49 A wall or a chair and do five
1:52 movements for each leg, five climbs
1:55 for each leg totaling ten, rest a

2:00 minute and do the series of
2:05 exercises again. This is a very
2:08 Important movement where you will train to
2:10 get up and sit down from a chair or bed
2:13 or from an armchair. you can do it
2:16 in two ways: without arm support
2:18 Like the video on the left and with the support
2:21 of the arms as in the video
2:23 Right. Make ten movements Rest one
2:27 minute and then repeat again
2:33 This is the same movement from another angle
2:36 Do it slowly, always breathing. Placing air
2:39 through the nose and releasing it through the
2:42 mouth. Everything very slowly
2:46 This is the cervical rotation movement
2:49 You will look one
2:51 side and to the other. You will count five times
2:55 for each side. Totaling ten. Rest
2:59 one minute and then repeat the movement or
3:02 perform within your capacity being able to
3:05 do a little less or a little more.
3:13 This is the movement of looking
3:16 up and down. Make five movements
3:19 looking up five movements
3:21 looking down Rest a minute and
3:24 then repeat
3:30 This is the movement of strengthening of
3:32 the calf muscles.
3:34 The calf plantar flexion movement.
3:36 You are going to be on the tip of your toes
3:39 raising the heels. Make ten
3:42 Movements. Rest for a minute and then repeat.
3:50 This is the rotational movement of the
3:54 spine. Turn the torso to both sides
3:57 to the right and to the left five
4:00 times for each side. Rest one minute
4:04 and then do another set five times
4:07 for each side. Don't forget to
4:13 breathe and remember the exercises
4:16 should be done
4:18 from Monday to Friday every day, right?
4:21 And you should do before using the
4:25 electrostimulation cap.

© 2025 Caumo W et al. *JAMA Network Open*

4:26 Thank you and have a great
4:29 week.

Bullet Points of the Exercises

0:25 slide
•2 sets;
•10 repetitions in each set;
• Within your physical capacity;
•1-minute rest between each set,
•Comfortable position;
•Back support;
•Foot support, if needed.

0:47 slide
•Slow movement,
•Don't forget to breathe,
•Sit in a comfortable place.

1:07 slide
•Foot support,
•Sit comfortably.

1:10 slide
•Extend your leg, one at a time.

1:30 slide
•Find a place with enough space,
•Open your arms,
•Slowly and while breathing.

1:44 slide
•Thigh muscle strengthening,

© 2025 Caumo W et al. *JAMA Network Open*

•Hip flexion movement,
•Use a wall or a chair support,
•Slow and unhurried movement.

2:09 slide
•Sit down and stand up from the chair,
•Without using your arms;
•Using your arms for support.

2:34 slide
•Stand up and sit down,
•Slowly,
•Always breathing

2:50 slide
•Cervical rotation movement,
•Perform within your physical capacity.

3:15
•Head flexion and extension,
•Slow movement
•Perform within your capacity.

3:31
•Plantar flexion,
•Calf strengthening,
•Slow movement,
•Perform within your capacity.

3:54
•Trunk rotation movement,
•Turn your torso to both sides,
•Slowly,
•Don’t forget to breathe.

© 2025 Caumo W et al. *JAMA Network Open*
