# Supplementary material for: Home-Based Transcranial Direct Current Stimulation vs Placebo for Fibromyalgia: A Randomized Clinical Trial
Source: JAMA Netw Open. 2025 Jun 6;8(6):e2514262. doi: 10.1001/jamanetworkopen.2025.14262 (PMC12144624; doi:10.1001/jamanetworkopen.2025.14262)
Supplement: Supplement 4. — Data Sharing Statement [file jamanetwopen-e2514262-s004.pdf]

Data Sharing Statement

Caumo. Home-Based Transcranial Direct Current Stimulation vs Placebo for Fibromyalgia.
JAMA Netw Open. Published June 06, 2025. doi:10.1001/jamanetworkopen.2025.14262

Data
Additional Information: NCT05845528
Data available: Yes
Data types: Deidentified participant data
How to access data: data available under request (wcaumo@hcpa.edu.br)
When available: With publication

Supporting Documents
Document types: None

Additional Information
Who can access the data: Who can access the data: Anyone requesting the data.
Types of analyses: Types of analyses: For any purpose.
Mechanisms of data availability: Mechanisms of data availability: Without investigator support.
Any additional restrictions: No aaditonal restricitions
